# Gas-phase engineered gold-on-paper SERS substrates for quantitative thiabendazole sensing in real matrices

**DOI:** 10.1007/s00604-026-08230-0

**Published:** 2026-07-01

**Authors:** Maher Darwish, Viktória Horváth, Hanan Mohammad, Gábor Katona, Judit Kopniczky, Zsolt Geretovszky, Attila Kohut

**Affiliations:** 1 Department of Optics and Quantum Electronics, University of Szeged, Dóm sq. 9, Szeged, 6720 Hungary; 2 Department of Pharmaceutical Chemistry and Drug Control, Faculty of Pharmacy, Wadi International University, Homs, Syria; 3 Institute of Pharmaceutical Technology and Regulatory Affairs, Faculty of Pharmacy, University of Szeged, Szeged, H-6720 Hungary

**Keywords:** Surface-enhanced Raman scattering, Spark ablation, Aerosol nanoparticle deposition, Thiabendazole, Real matrices, Plasmonic interface

## Abstract

A three-dimensional (3D) Au nanoparticles (AuNPs)-cellulose interface was engineered via solvent-free spark ablation and direct particle deposition onto filter paper, forming a percolated junction network with abundant nanoscale gaps that enables surface-enhanced Raman scattering signal amplification under a fiber-optic probe geometry. The resulting 3D plasmonic network exhibits good signal reproducibility, as verified by Rhodamine 6G mapping (RSD 13.5–16.6%). Using thiabendazole (TBZ) as a representative small-molecule residue, the platform enables quantitative detection down to 0.08 ppm in methanol and 0.10 ppm in untreated apple juice, supporting residue monitoring in real matrices. A nonlinear logarithmic calibration model captures the concentration-dependent response without requiring complex chemometric processing. TBZ-spiked apple juice samples showed satisfactory recoveries of ca. 83–105%, while selectivity against thiram, batch-to-batch repeatability, and intra-/inter-day precision further confirm the analytical robustness of the engineered interface. Overall, the AuNP-cellulose junction network couples interface-driven signal amplification with point-of-need applicability, advancing flexible Raman-based sensing for rapid residue screening in food safety and related chemical/biosensing scenarios.

**Graphical Abstract:**

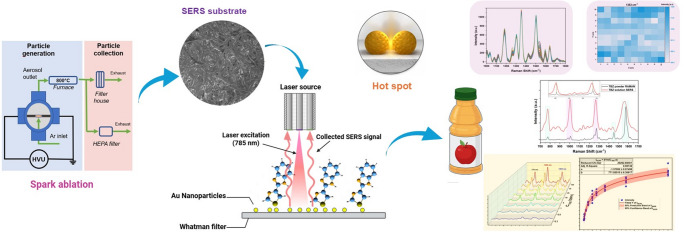

**Supplementary Information:**

The online version contains supplementary material available at 10.1007/s00604-026-08230-0.

## Introduction

The widespread agricultural application of thiabendazole (TBZ), a systemic benzimidazole fungicide, has raised significant food safety concerns due to its persistence in food items and potential adverse health outcomes, including endocrine disruption and teratogenic effects [1]. Consequently, regulatory authorities such as the US-EPA and EFSA have established stringent maximum residue limits (MRLs) for TBZ, typically ranging from 0.01 to 10 mg/kg depending on the agricultural product [2, 3]. Enforcing these regulations predominantly relies on chromatographic methodologies (e.g., HPLC and GC-MS). While highly sensitive, these conventional techniques suffer from severe limitations for on-site monitoring, as they demand comprehensive sample preparation, advanced instrumentation, and highly skilled personnel [4]. Such challenges underscore the pressing need for alternative analytical strategies that are sensitive, cost-effective, and field-deployable.

Among emerging techniques, surface-enhanced Raman spectroscopy (SERS) has risen to prominence as a potent analytical tool for the trace identification of chemical residues, including pesticides like TBZ [5]. SERS exploits the phenomenon of localized surface plasmon resonance (LSPR) generated by metallic nanoparticles (typically gold or silver), which results in a massive amplification of the electromagnetic field. Consequently, the performance of any SERS-based application is fundamentally governed by the engineered nano-interface where the analyte interacts with the plasmonic substrate [6, 7]. Recently, the development of flexible SERS platforms, particularly paper-based substrates, has attracted considerable interest. Paper offers natural porosity that allows for the dense deposition of metal nanoparticles, promoting the formation of closely packed clusters. These clusters serve as three-dimensional interfaces rich in sub-10-nm gaps (“hot spots”), which are crucial for interface-driven signal amplification. Furthermore, paper substrates are lightweight, affordable, and biodegradable, aligning perfectly with the rising demand for sustainable, point-of-care analytical devices [8].

A wide variety of paper-based SERS substrates have been realized by drop-casting colloidal nanoparticles onto cellulose filters or office paper, as well as by inkjet printing metal nanoparticle inks. In these approaches, the plasmonic nanoparticles are typically stabilized by capping agents such as citrate, cetyltrimethylammonium bromide, polyvinylpyrrolidone, or polymer matrices, which are essential for colloidal stability but can partially shield the metal surface and limit direct analyte access to high-field junctions. In addition, drop-casting and printing predominantly yield surface-confined nanoparticle layers, whereas the underlying fibrous network remains largely unoccupied, so that only a fraction of the available paper volume contributes to the formation of electromagnetic hot spots [9, 10].

By contrast, gas-phase spark ablation produces ligand-free metal nanoparticles with exceptionally clean surfaces and well-defined plasmonic properties, without the use of solvents, reducing agents, or surfactants [11, 12]. When combined with filtration-based collection, these nanoparticles are driven into and immobilized throughout the three-dimensional cellulose network, creating a percolated AuNP-cellulose interface in which hot spots are distributed within the accessible depth of the porous fibrous network rather than being restricted to the outer surface. This interface architecture conceptually resembles filter-SERS strategies that exploit filtration to co-concentrate nanoparticles and analytes in porous membranes, but here the plasmonic network is generated entirely by a physical aerosol route. As a result, the engineered AuNP-cellulose interface combines a chemically clean, ligand-free metal surface with a volumetric hot-spot distribution tailored to fiber-based excitation geometries [13].

Spark ablation involves repetitive high-voltage sparks between metal electrodes, generating a transient plasma that vaporizes the electrode material and condenses it into nanoscale aerosol particles. Beyond eliminating wet chemistry, this physical route is inherently compatible with continuous aerosol processing, which makes it attractive for scalable SERS substrate fabrication. When the gas-borne nanoparticles are collected by filtration into flexible cellulose membranes, they form an optically robust plasmonic layer deep in the filter without any post-deposition chemical functionalization. In this study, we translate our earlier spark-ablation paper-SERS concept into a quantitatively validated analytical platform for TBZ detection. Specifically, solvent-free AuNPs were directly aerosol-deposited onto cellulose filter microfibers to create a three-dimensional AuNPs-cellulose interface with abundant plasmonic junctions and without wet-chemical synthesis, capping ligands, or surface functionalization [14]. Beyond substrate fabrication, the work establishes an analytical workflow for TBZ quantification in methanol and untreated commercial apple juice using a simple nonlinear calibration model and ICH-guided validation. The study therefore positions gas-phase engineered gold-on-paper SERS substrates as practical sensing platforms for rapid residue screening in real matrices.

## Experimental section

### Nanoparticle generation and substrate fabrication

Materials, reagents and instrumentation are provided in the Electronic Supplementary Material (ESM). For the fabrication of the plasmonic interfaces, a spark discharge nanoparticle generator was used, which was in a setup similar to the one introduced in our group’s previous work (a detailed schematic representation of the fabrication setup is provided in Figure S1 in the ESM) [14]. A stainless-steel 6-way KF-40 cross (QF40-150-6X; Kurt J. Lesker, USA) was utilized as a spark chamber housing a pair of gold electrodes (99.9% purity, Goodfellow Cambridge Ltd., Cambridge, UK), positioned horizontally 1 mm apart from each other, with the connections for the gas inlet and aerosol outlet aligned perpendicular to the electrodes. Argon (99.996% purity, Messer Hungarogáz Ltd., Budapest, Hungary) was used as the carrier gas, with a volumetric flow rate of 10 L.min^− 1^ (mass flow controller model GFC16, Aalborg Inc., New York, NY, USA). Sparking was maintained using a power supply designed for high-frequency spark ablation experiments (HVU, VSParticle B.V., Delft, The Netherlands), with the charge voltage set to 900 V and the spark frequency to 2000 Hz across all experiments. After leaving the spark chamber, the aerosol containing the nanoparticles was led through a tube furnace preheated to 800^°^C for compaction (EHA 12/300B, Carbolite Gero GmbH, Neuhausen, Germany).

The active SERS substrates were subsequently fabricated by passing the compacted aerosol directly through a porous paper filter membrane (1001-047 Whatman) for 7 min. This direct deposition process effectively embedded the nanoparticles within the fibrous matrix, creating a robust, three-dimensional plasmonic network. The nanoparticle-loaded filters were cut into pieces of 6 × 6 mm^2^ for mapping experiments and 4 × 4 mm^2^ for routine SERS measurements.

### Preparation of TBZ standard and untreated commercial matrix

In this work, all solutions were prepared in methanol, which consisted of the R6G probing molecule, thiram, as a probable contaminant, and the target analyte, TBZ. A range of standard solutions of TBZ was created in methanol, with concentrations spanning from 0.1 to 100 ppm.

To accurately reflect real-world applications and provide a more authentic assessment of TBZ behavior in a typical environment, TBZ was directly introduced into apple juice (sourced from a local market), ensuring the final concentrations matched those of the standard. For the SERS analysis, aliquots of 3 µL from each TBZ solution were applied to the AuNPs@filter substrate piece. The samples were left to dry completely for a few minutes at ambient temperature, allowing the analyte molecules to thoroughly interact with the plasmonic interface before proceeding with the spectral measurements.

### SERS measurements and data processing

Raman and SERS spectra were obtained using an Ocean Optics QE65000 Raman Spectrometer. This instrument featured a thermoelectrically cooled charge-coupled device (CCD) detector, which was regulated at −10 °C. A 785 nm wavelength excitation laser (LASER-785-LAB-FC, Ocean Optics Inc.) with a laser power of 15 mW (measured at the probe tip) was utilized. The spectrometer and the excitation laser were coupled to a fiber-optic Raman probe (AvaRaman-PRB-785, Avantes B.V., The Netherlands) through standard fiber optic connectors (SMA and FC/PC respectively). The Raman probe features a fixed working distance of 7.5 mm and delivers the laser beam through a central fiber surrounded by collection fibers. The laser spot size at the focal plane was calculated to be approximately 100 μm in diameter, corresponding to an illuminated area of ~ 7.85 × 10^− 3^ mm^2^. The substrate was positioned perpendicular to the probe axis using an adjustable two-dimensional sample holder, ensuring that the laser focus coincided with the nanoparticle-decorated filter surface. This relatively large laser spot ensures that each SERS measurement samples multiple fiber-nanoparticle interaction zones, providing spatial averaging that stabilizes signal variability arising from microscopic substrate heterogeneity. To minimize probe-to-sample variability, a constant length spacer was utilized during the measurements. The spectra were recorded over a Raman shift range of 200 to 2800 cm^− 1^, with a spectral resolution of approximately 5–6 cm^− 1^. To ensure reliable results and account for variations within the samples, SERS measurements were performed at eight distinct locations on each sample. Each spectrum was typically acquired over a duration of 120 s.

Given the intrinsic variability present in SERS measurements, the raw spectra were not employed directly for model development. This was due to the presence of issues like baseline fluctuations and random noise, which could negatively impact the accuracy of prediction and classification models. Consequently, data preprocessing techniques were implemented to enhance the quality of the spectra and strengthen the robustness of the models [15, 16]. The spectral analyses were carried out utilizing SpectraGryph 1.2 spectroscopy software, adhering to a methodical three-step preprocessing protocol. The first step, spectral averaging, aimed to reduce variability between data points by averaging the spectra obtained from eight distinct locations for each sample, resulting in a representative final spectrum. The second step involved noise reduction, where a Savitzky-Golay filter was applied with a smoothing interval of 10 and a polynomial order of 3. This technique effectively reduced high-frequency noise while maintaining the integrity of the spectral peaks. Lastly, baseline correction was implemented using an adaptive approach with a coarseness parameter set at 7, which served to remove background interference and improve the resolution of the spectral peaks.

In order to determine the relationship between Raman intensity and TBZ concentration, multiple fitting models were assessed utilizing OriginPro software. The efficacy of each model was analyzed through statistical goodness-of-fit metrics, such as R² values and residual analysis, to identify the most appropriate mathematical model for quantitative prediction.

## Results and discussion

### Substrate characterization and interface morphology

**Fig. 1:**
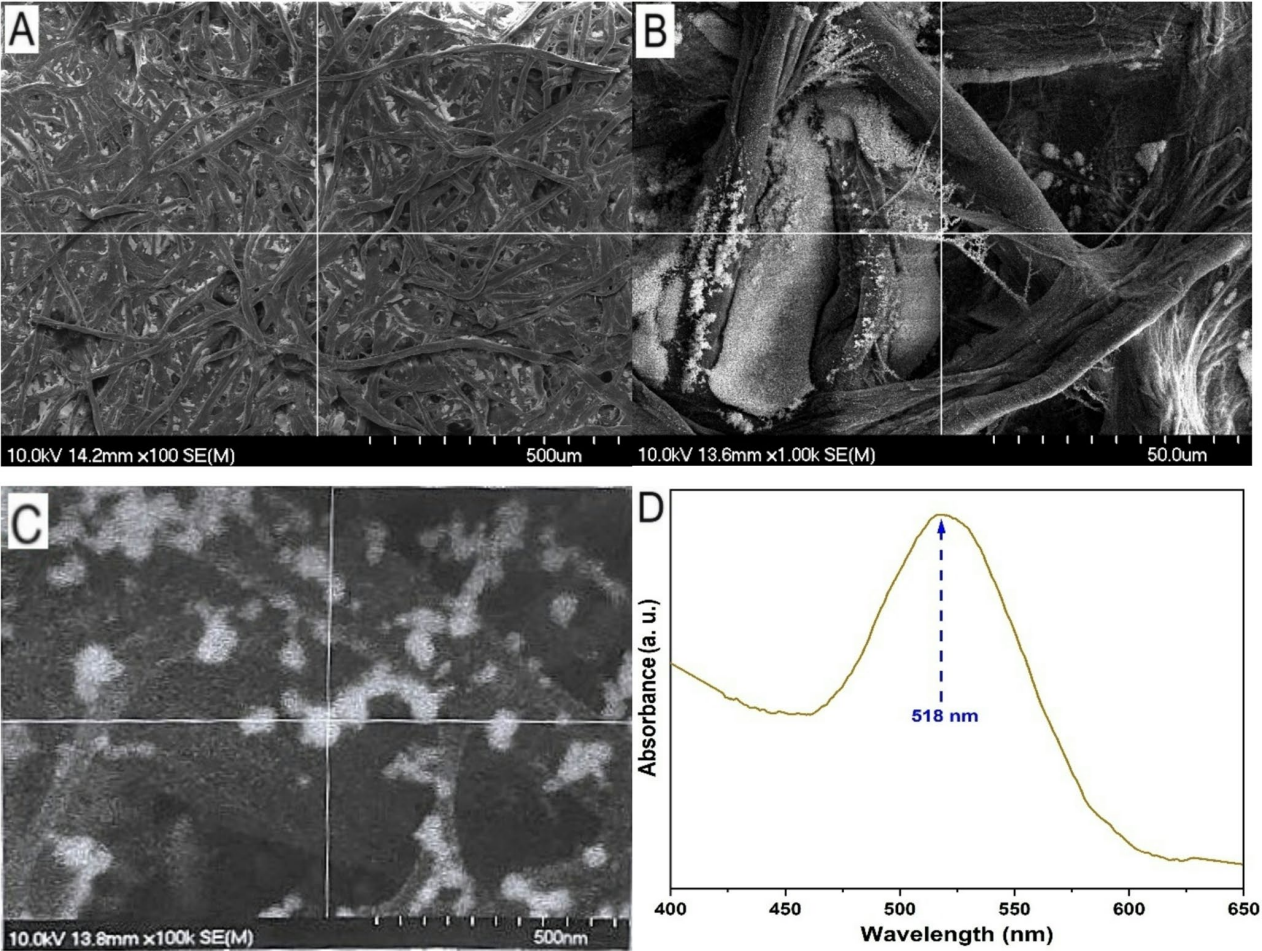
(**A**-**C**) SEM images of the AuNPs@filter substrate at increasing magnifications, showing gold nanoparticles distributed among cellulose fibers, and (**D**) UV-Vis absorption spectrum of the AuNPs@filter substrate

In order to fabricate SERS substrates, spark ablation-generated Au NPs were collected by paper filters according to the details given in the Experimental Section. The SEM micrographs shown in Fig. 1 illustrate the results of the fabrication process. At the lowest magnification (Fig. 1A), the intricate three-dimensional fibrous framework of the Whatman 1001-047 filter is well exemplified. This architecture, characterized by interwoven cellulose microfibers with diameters of 20–30 μm and inter-fiber pores ranging from 5 to 20 μm, provides a high surface area and enables uniform deposition of the 3 µL analyte droplets. The porous structure also facilitates deep penetration of gold nanoparticles into the filter matrix, forming a percolating plasmonic network rather than a surface-only coating. This three-dimensional interface is crucial for achieving high interaction volumes between the analyte and the plasmonic hotspots. In these SEM images (cf. Figure 1), the nanoparticles are characterized as bright entities due to their higher atomic number, resulting in a pronounced contrast against the cellulose fibers, and are predominantly deposited in between the fibers, forming clusters of varying density. This can be seen in Fig. 1B, which shows a large nanoparticle agglomerate in the intersection of different fibers with only relatively few particles deposited on the surface of the fibers. A higher magnification image (Fig. 1C) shows that the nanoparticles are mostly near-spherical in shape due to the heat treatment described in the Experimental section and having sizes approximately ranging from 20 nm to 50 nm, which is in line with the typical size distribution of plasmonic aerosol nanoparticles generated under similar conditions (please see Figure S2 in the ESM).

The SEM micrographs indicate that the gold nanoparticles are not deposited as a smooth or continuous metallic layer on the filter surface. Instead, they appear as discontinuous clusters distributed across the cellulose fiber network, with locally varying particle densities in different regions of the substrate. This heterogeneous distribution is expected for aerosol deposition on porous fibrous materials, where the gas-borne nanoparticles follow the carrier-gas flow through the open pore structure and are captured at accessible fiber surfaces, fiber intersections, and inter-fiber voids. Regions with higher local particle density are therefore mainly observed at geometrically favorable collection sites, such as fiber junctions, where the airflow path, available surface area, and local confinement promote particle accumulation. The primary interaction mechanisms between these nanoparticles and the cellulose fibers can be identified as physical adsorption, where the particles adhere to the surface, and mechanical entrapment, where they are physically wedged within the fibrous network. These interactions are probably enhanced by the presence of hydroxyl groups on the cellulose fibers, enabling them to interact with the nanoparticles via van der Waals forces and hydrogen-bonding interactions, as commonly reported for cellulose-supported plasmonic SERS substrates [17, 18]. Although the distribution and particle size are not entirely uniform, AuNP-rich domains are repeatedly observed across the examined regions, increasing the accessible plasmonic surface area while preserving the structural integrity of the cellulose substrate. Importantly, this combination of physical adsorption and mechanical entrapment stabilizes AuNP clusters at fiber junctions, thereby promoting the formation of nanoscale interparticle junctions that act as SERS-active hot spots across the AuNP-cellulose network.

AFM topography images in Figure S3 further support the heterogeneous AuNP–cellulose morphology. The 20 × 20 µm^2^ scan reveals rough, corrugated AuNP-decorated domains with fine-scale protrusions consistent with aggregated AuNP-rich regions. Such clustered AuNP arrangements are relevant for SERS because interparticle junctions generate electromagnetic hot spots, and similar AuNP-loaded paper substrates have shown that aggregate size and surface coverage strongly influence SERS performance. Together with SEM, the AFM data support a discontinuous clustered AuNP-cellulose network indicating the presence of inhomogeneities on the microscopic level. The effects of these inhomogeneities on the macroscopic SERS response of the substrates will be discussed later in light of Raman mapping results. The UV-Vis absorption spectrum of the fabricated SERS substrates, shown in Fig. 1D, exhibits a distinct LSPR peak at around 518 nm with a full width at half-maximum (FWHM) of approximately 67 nm, which is characteristic of small, spherical gold nanoparticles [19].

### SERS performance test of the engineered interfaces

**Fig. 2:**
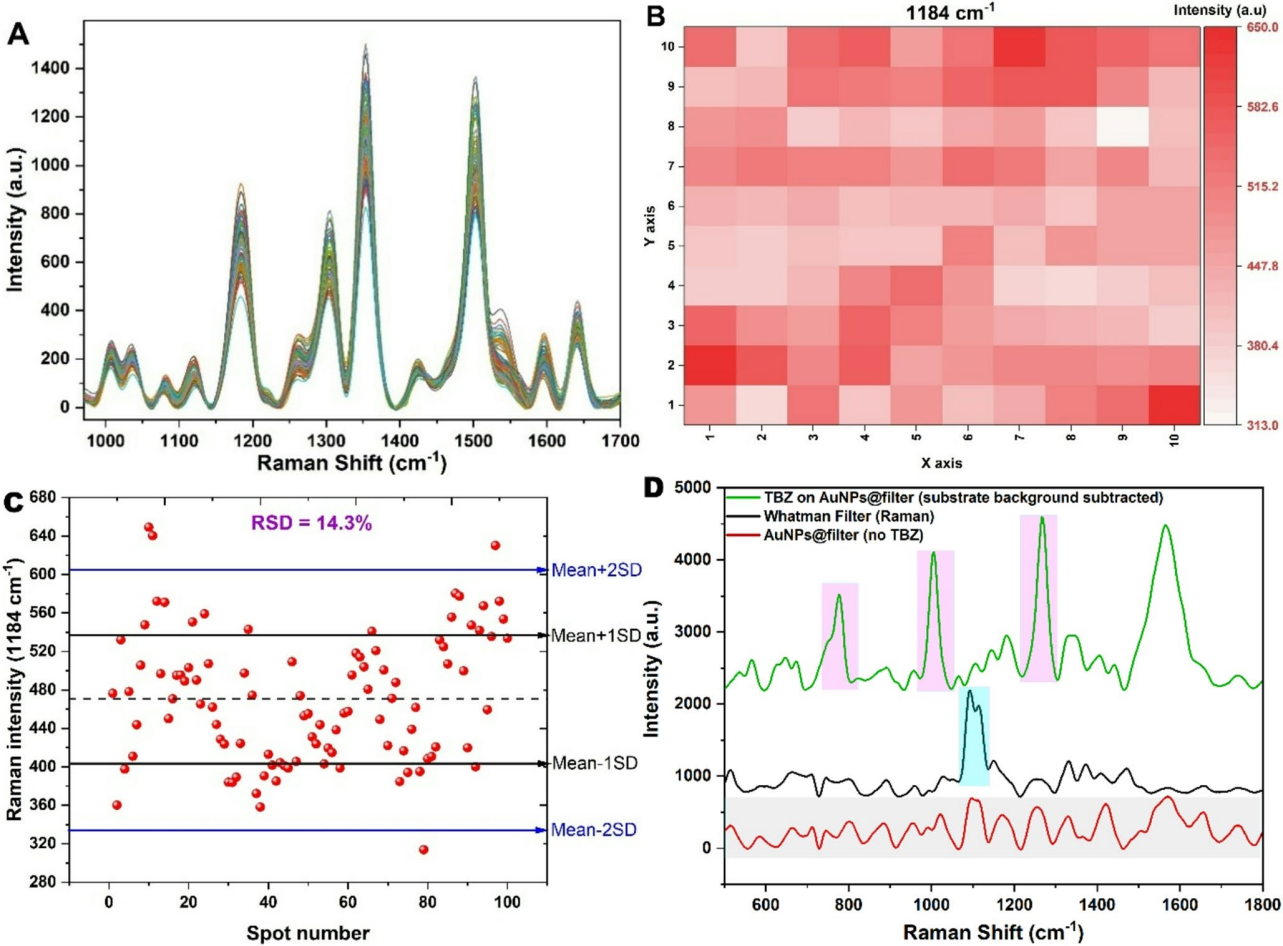
(**A**) R6G SERS spectra on AuNPs@filter. (**B**) Spatial SERS map of the substrate (R6G). (**C**) Intensity scatter plot from the 1184 cm^−1^ R6G band. (**D**) Background/matrix assessment: AuNPs@filter (red), bare Whatman paper (black; cellulose at 1093 and 1114 cm^−1^), and AuNPs@filter + 10 ppm TBZ (green) showing TBZ peaks at 776, 1004, and 1265 cm^−1^ with negligible substrate interference

In this work, the electromagnetic performance of the substrates is evaluated experimentally through their LSPR response, SERS mapping, and analytical enhancement factor (in the ESM), rather than by numerical field simulations. Therefore, before the actual use of the synthesized AuNPs@filter substrate, a proof-of-concept experiment was conducted to evaluate its performance as a signal-amplifying interface. This assessment involved the use of R6G as a model Raman reporter molecule to test the effectiveness of the developed substrate [20]. To conduct a thorough evaluation of the substrate, an analysis of the spatial homogeneity and uniformity of the SERS-active surface was performed using Raman mapping techniques. A systematic scan encompassing a total area of 6 × 6 mm^2^ – representing a single substrate – was carried out with a step size of 0.5 mm, yielding 100 individual measurement points across an approximately 5 × 5 mm^2^ region. Measured spectra are shown in Fig. 2A. The SERS spectrum acquired from a 100 µM solution of R6G for 30 s integration time reveals four identifiable peaks, each representing particular vibrational modes of the R6G molecule. The peak at 1184 cm^− 1^ is associated with C-H in-plane bending. The peak at 1304 cm^− 1^ corresponds to C-O-C stretching vibrations. Additionally, the peaks at 1353 cm^− 1^ and 1502 cm^− 1^ arise from C = C stretching vibrations within the benzene ring [21].

The intensity distribution of the four characteristic R6G peaks was systematically measured across the substrate surface using Raman mapping (Fig. 2B and Figure S4). To quantitatively evaluate the spatial reproducibility of the substrate, the relative standard deviation (RSD%) for each of the four significant peaks was calculated from the 100 measurement points. The RSD% values for the characteristic peaks were found to be in the range of 13.5% to 16.6%, with approximately 68% of the measured intensities falling within ± 1 standard deviation (SD) and ~ 95% within ±2SD limits, consistent with the normal distribution (Fig. 2C and Figure S5).

In addition to the RSD analysis, the 100-point R6G mapping dataset was further examined using quadrant-wise statistics by dividing the 10 × 10 map into four equal regions. For the 1184, 1304, 1354, and 1502 cm^− 1^ bands, no statistically significant quadrant effect was observed (one-way ANOVA *p* = 0.639, 0.129, 0.333, and 0.226, respectively; Kruskal–Wallis *p* > 0.05 in all cases), indicating that the intensity variation is predominantly local/microscopic rather than a large-area systematic spatial bias across the substrate. Descriptive statistics for the mapping dataset are provided in Table S1. This level of variability is consistent with the microscopic heterogeneity inherent to the paper-based substrate, where the three-dimensional fibrous network leads to a non-uniform distribution of gold nanoparticle clusters. While this raw variability is higher than that of highly ordered, lithographically-defined substrates, it is an expected and acceptable characteristic for a field-deployable, paper-based SERS platform fabricated via a simple, single-step, solvent-free method. Further optimization of the deposition protocol and substrate design is expected to reduce this variability if needed for specific applications. Crucially, the high analytical reliability of the method is maintained through physical spatial averaging. As detailed in Sect. 2.3, the fiber-optic Raman probe utilizes a relatively large laser spot size (~ 100 μm). This spot size is significantly larger than the characteristic dimensions of the nanoparticle clusters, ensuring that each measurement integrates the signal from multiple plasmonic hot spots within the interface. This physical averaging effect effectively stabilizes the analytical signal, demonstrating that the substrate is robust for practical quantitative analysis despite its microscopic roughness.

A critical concern with paper-based SERS substrates is the potential interference from the cellulose matrix. To evaluate this, we characterized the Raman spectrum of the bare Whatman 1001-047 filter paper, which exhibits characteristic cellulose peaks at 1093 cm^− 1^ and 1114 cm^− 1^ (C–O–C stretching vibrations) with modest intensity. Notably, after AuNP deposition, these cellulose peaks become undetectable (Fig. 2D), indicating that the Au nanoparticles effectively mask the underlying paper substrate. This is advantageous for sensing applications as it eliminates potential spectral overlap and ensures that the observed SERS signals arise predominantly from the target analyte interacting with the plasmonic interface.

### Detection of TBZ by the engineered interfaces

**Fig. 3:**
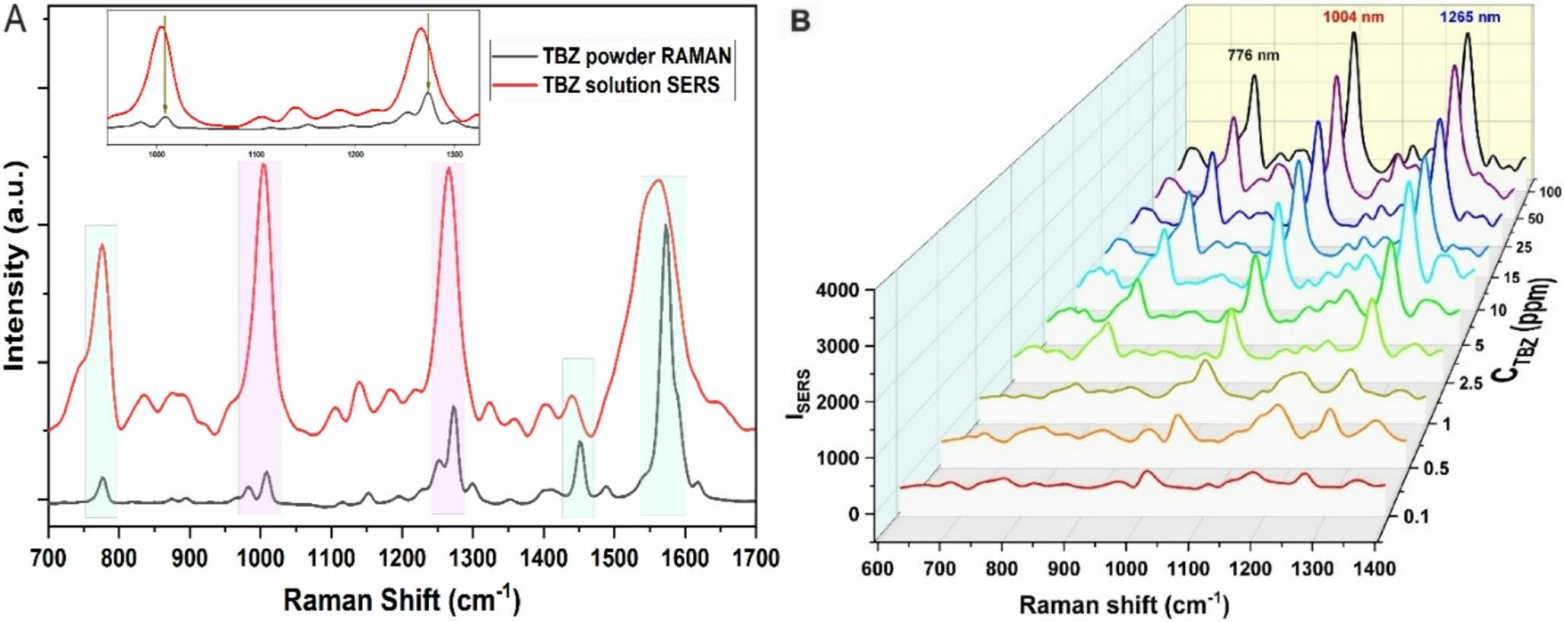
(**A**) SERS spectrum of 100 ppm TBZ in methanol on AuNPs@filter (red) compared with the conventional Raman spectrum of solid TBZ (black); intensities are shown on different scales. (**B**) Concentration-dependent SERS spectra of TBZ in methanol (100–0.1 ppm) acquired on AuNPs@filter

The engineered AuNPs@filter SERS interfaces were employed to detect TBZ. Figure 3A presents the SERS spectrum of TBZ in methanolic solution (100 ppm), alongside with the conventional Raman spectrum of solid TBZ powder recorded on a non‑enhancing glass slide. The powder spectrum serves as a reference for assigning the intrinsic vibrational modes of TBZ, while the SERS spectrum reveals the surface‑induced modifications that occur upon adsorption onto the AuNPs within the plasmonic hot spots.

The well-defined characteristic peaks of TBZ are displayed clearly at 776, 983, 1008, 1275, 1452, and 1573 cm^− 1^. Among these, two particularly strong and significant peaks at 776 and 1008 cm^− 1^ are related to the out-of-plane C–H bending modes of the TBZ molecular ring. In contrast, the peaks at 1452 and 1572 cm^− 1^ arise from simultaneous stretching vibrations of the C = N. Furthermore, the peak at 1275 cm^− 1^ was attributed specifically to the stretching vibration of the entire ring structure of the TBZ molecule, while the peak located at 982 cm^− 1^ was determined to be caused by the stretching of the C–S bond within the molecule [22, 23]. The most prominent peak located at 1572 cm^− 1^ in the SERS spectrum of TBZ dissolved in methanol exhibits a notable broadening compared to other peaks. This broadening is primarily ascribed to the pronounced influence of the methanol signal, which overlaps with the TBZ spectrum [24]. The remaining peaks can directly be attributed to the TBZ molecule, with minimal interference considered.

The SERS spectrum of TBZ in a methanol solution reveals significant spectral shifts and intensity variations, that lend insight into its adsorption mechanism on the gold nano-interface. Surface chemistry plays a central role here, governing the preferential interfacial localization of TBZ on the exposed Au surface via N/S coordination, while the Au nanostructure provides the dominant plasmonic amplification once the analyte is adsorbed within nanometric proximity to the metal surface. Specifically, the peak at 1008 cm^− 1^ shifts to 1004 cm^− 1^ with a relative enhancement in intensity compared to other peaks, which is consistent with a surface-induced electronic perturbation and a possible charge-transfer contribution involving the heterocyclic N and/or S atoms of TBZ, plausibly affecting C = N-associated vibrations. In a similar fashion, the peak at 1275 cm^− 1^ transitions to 1265 cm^− 1^, suggesting a surface-induced stabilization of heteroaromatic ring vibrations (C = N-rich modes) as TBZ coordinates to Au via its N/S site. The enhanced intensities observed at 776, 1008, and 1275 cm^− 1^ are consistent with an adsorption geometry in which out-of-plane ring modes are preferentially enhanced, plausibly reflecting a tilted-to-near-perpendicular orientation of the TBZ framework relative to the local Au surface, where the benzimidazole-thiazole framework couples efficiently to the local plasmonic field, amplifying out-of-plane ring vibrations [23, 25]. This interpretation corroborates findings from prior studies that identified robust interactions through the thiazole moiety, indicating a preference for sulfur as the primary binding site. Research by Kim et al. and Oliveira et al. underscores the essential role of the sulfur atom in the adsorption of TBZ onto metallic surfaces. While Kim et al. reported that TBZ predominantly engages with silver surfaces through π-electron interactions under neutral conditions, they also noted that in acidic environments, adsorption occurs via sulfur and nitrogen atoms, resulting in a tilted orientation [26]. Furthermore, Oliveira et al. illustrated that the anchoring of TBZ on silver nanoparticles (AgNPs) is mediated by sulfur, as theoretical computations reveal that interactions involving sulfur significantly alter the electronic structure and optical absorption spectrum, aligning with empirical SERS observations. Additionally, the selection rules for SERS indicate that vibrational modes oriented perpendicularly to the metallic surface exhibit stronger enhancement, thereby supporting the hypothesis of a perpendicular molecular orientation of TBZ, which maximizes its interaction with the plasmonic field [27]. Overall, the observed spectral shifts and relative intensity changes suggest a strong interfacial interaction between TBZ and the AuNP surface, likely involving heterocyclic N/S sites, although the exact adsorption geometry and binding mechanism would require dedicated mechanistic studies for definitive confirmation. It is important to highlight (Fig. 2D), when TBZ (10 ppm) is applied to the AuNPs@filter interface the three diagnostic SERS peaks at 776, 1004, and 1265 cm^− 1^ are clearly resolved with adequate signal-to-noise ratios. These peaks fall in spectral regions completely free from cellulose interference, with the nearest paper peak (1093 cm^− 1^) being > 90 cm^− 1^ away and suppressed by the Au coating. Importantly, the TBZ peak at 1004 cm^− 1^, used for quantitative analysis, shows no baseline contribution from the paper substrate, highlighting the efficiency of the interface-driven signal amplification. In this architecture, the cellulose fibers mainly act as a porous three-dimensional support that promotes AuNP distribution, analyte retention, and analyte transport to plasmonically active sites [28].

### Quantitative TBZ detection

In order to test our AuNPs@filter SERS platform’s performance for the detection of TBZ, the surface-enhanced Raman signal of the analyte molecule was measured at systematically varied concentrations. As illustrated in Fig. 3B, the SERS intensity of TBZ peaks exhibited a progressive decrease correlating with the decrease of the pesticide concentration, particularly for the most prominent peaks at 776, 1004, and 1265 cm^− 1^. The latter two peaks remained distinctly identifiable even at 0.1 ppm, the minimal concentration tested in the present study. The peak corresponding to 1004 cm^− 1^ exhibited both the greatest intensity and the lowest detection limit; thus, it was utilized to formulate quantitative calibration curves linking SERS intensity to TBZ concentration.

**Fig. 4:**
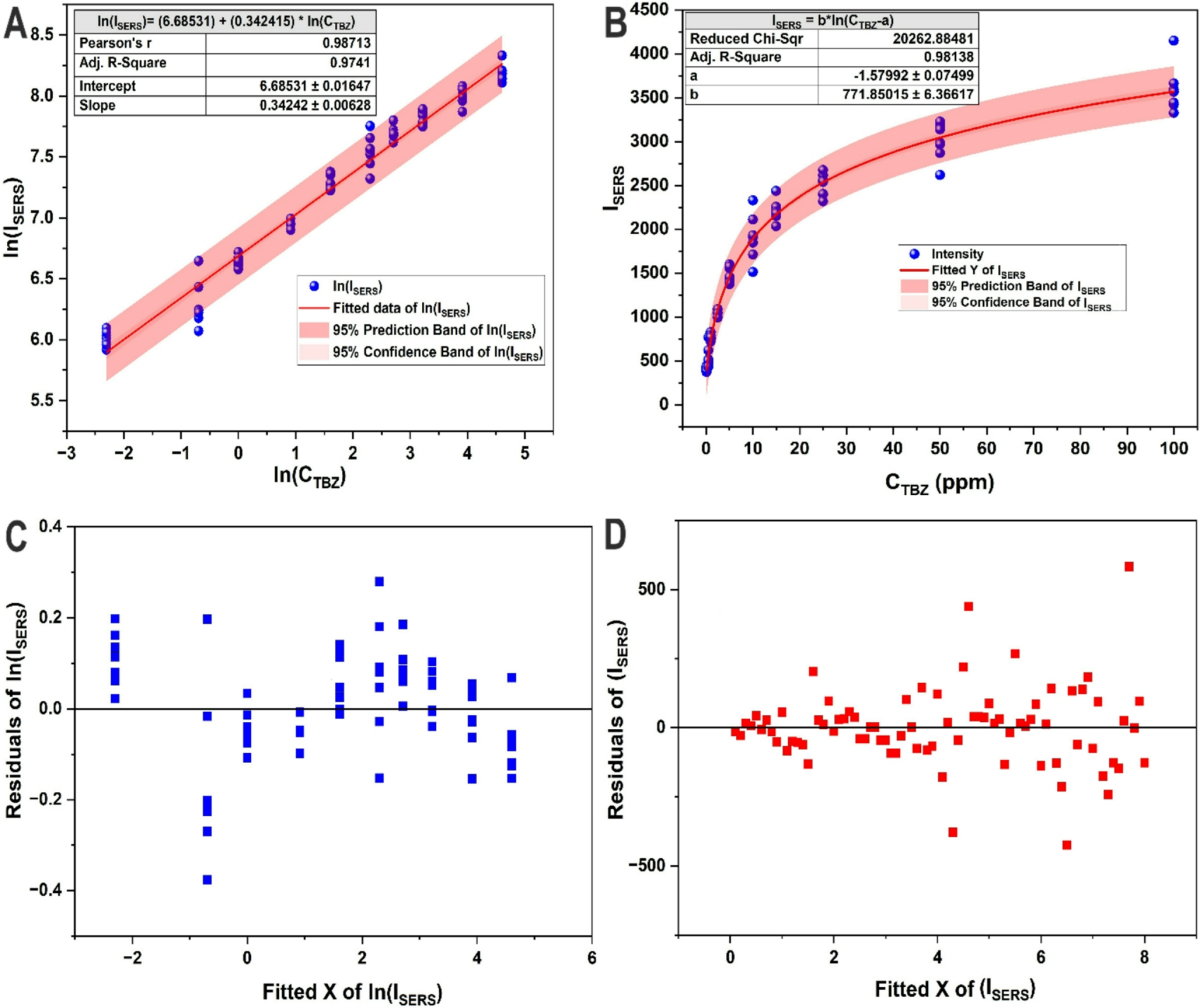
(**A**) Double-ln calibration plot showing linearized fit, (**B**) nonlinear logarithmic model fit, and residuals plot for (**C**) linearized and (**D**) nonlinear models

To evaluate the efficacy of the fitting process, we compared several widely acknowledged linear or linearized fitting models including linear regression, single logarithmic, and double-logarithmic models as well. Among them, the double logarithmic representation showed the most favorable performance as shown in Fig. 4A, where the natural logarithm of the measured SERS intensity followed a linear trend as a function of the natural logarithm of the TBZ concentration. Although, in general, a linear calibration curve is desirable in most analytical techniques, we also evaluated nonlinear models to determine whether calibration accuracy could be further improved. Our evaluation indicated that the nonlinear logarithmic model $\:{I}_{SERS}=b\mathrm{ln}\left({C}_{TBZ}-a\right)$ yielded the best empirical fit among the tested models. In this model, *b* describes the sensitivity of the SERS response to changes in the interfacial TBZ population, whereas *a* acts as a concentration offset that shifts the onset of measurable signal and can be interpreted as an effective threshold term. It should be emphasized that the model is used here as an empirical calibration function for this substrate-analyte system, rather than as a universal mechanistic adsorption equation.

Accordingly, we performed a comparative analysis between this nonlinear logarithmic model and the previously identified linearized model to determine which provided a more accurate representation of the data. As shown in Fig. 4B, the nonlinear fit exhibited a higher R^2^ value of 0.981, whereas the linearized model gave a slightly lower R^2^ value of 0.974 (cf. Figure 4A). While both values indicate good fits, the nonlinear model ultimately demonstrated better performance.

Analyzing the residuals for each model yields additional insights into their fitting quality. As demonstrated in Fig. 4 (C, D), the residual plot for the nonlinear model reveals a more random and uniformly distributed pattern around the zero line, which suggests a superior fit without evident systematic bias. Conversely, the residuals associated with the linearized model reveal significant clustering and potential patterns, indicating that it does not comprehensively capture the data behavior. These findings are consistent with the higher R² value and enhanced predictive performance of the nonlinear model, reinforcing its appropriateness for analyzing this dataset. A comparative analysis was conducted between the predicted values from both models (Predicted C) and the actual values (Real C), as presented in Table S2. The results indicate that the nonlinear model consistently delivered predictions that aligned more closely with the actual values across all evaluation points. For example, when Real C equaled 0.1 ppm, the nonlinear model yielded a prediction of 0.109 ppm, in contrast to the linearized model prediction of 0.136 ppm. Likewise, at Real C of 100 ppm, the nonlinear model prediction was 101 ppm, while the linearized model prediction fell to 78.9 ppm. This enhanced predictive capability further emphasizes the applicability and reliability of the nonlinear model for accurate quantitative analysis over a wide dynamic range.

Although a linear response is often desirable in analytical practice, it is not necessarily expected over the full concentration range in SERS, because the measured intensity depends not only on bulk analyte concentration but also on adsorption, accessibility of plasmonically active sites, and the heterogeneous distribution of hot spots across the substrate. In simple idealized cases, adsorption-driven saturation may approximately follow a Langmuir model, but this requires more homogeneous and equivalent adsorption sites than are likely present in the current porous AuNP-cellulose interface. In SERS, linearity is often obtained only over a limited working range, whereas broader concentration ranges may show nonlinear behavior consistent with more complex adsorption and site-accessibility effects [29]. In the present system, the observed sub-linear response is more reasonably attributed to the integrated contribution of non-equivalent adsorption and enhancement environments within the 3D paper-based plasmonic network.

Although the calibration curve of quantitative TBZ detection by using our SERS substrates is best described by a nonlinear model, as shown above, the limit of detection (LOD) was determined following the internationally accepted IUPAC/ICH standard, to promote comparability:

$$\:LOD=3.3\times\:\frac{\sigma\:}{S}$$

Where S is the local (low-level) slope of the linear part (0.1 to 5 ppm) and σ is the standard deviation of the baseline signal. The baseline noise was first characterized by measuring ten independent blank SERS substrates (without TBZ) to determine the average SERS signal intensity and its standard deviation at the 1004 cm^− 1^ peak [30, 31]. This calculation yielded an LOD of 0.08 ppm (Figure S6), a value that is significantly lower than the MRLs for TBZ in food products, confirming the method’s high sensitivity and suitability for regulatory monitoring [32].

### Validation of the engineered nano-interfaces for TBZ detection

Method validation is a structured approach designed to verify that an analytical method is appropriate for its intended application. This process is vital for ensuring the quality, dependability, and uniformity of the results obtained. According to the International Council for Harmonization guidelines (ICH Q2(R2)), several critical parameters – such as precision, accuracy, specificity, robustness, linearity, and limit of detection (LOD) – need to be assessed. The subsequent paragraphs detail the key characteristics necessary for thorough validation [30].

Precision is defined as the extent of agreement among multiple measurements of the same sample conducted under controlled and identical conditions, which serves as an indicator of the reproducibility and reliability of an analytical methodology. In the present investigation, precision was evaluated through the examination of repeatability (intra-day precision) and intermediate precision (inter-day reproducibility) as detailed in Table 1 [33]. To determine intra-day precision, a 10 ppm (100% concentration) TBZ standard solution was analyzed, and TBZ spectra were collected from ten random sites on the AuNPs@filter substrate. The RSD%, of the Raman peak intensity at 1004 cm^− 1^ was computed to quantify the variability amongst the replicates. As shown in Figure S7 summarized in Table 1, this yielded an RSD% of 9.85%, which is well below the commonly accepted 25% RSD limit for SERS-based analytical methods, thereby demonstrating a substantial degree of repeatability [31, 34].

For inter-day precision, the same 10 ppm TBZ solution was analyzed using three independently prepared AuNPs@filter batches. The predicted concentrations across nine measurements showed an overall RSD of 2.34%, as summarized in Table 1 and illustrated in Figure S8. To statistically evaluate batch effects, one-way ANOVA was performed on the corresponding SERS intensities (*n* = 24), revealing no significant difference among batches, F(2, 21) = 1.33, *p* = 0.287. These results indicate good inter-batch reproducibility of the fabricated substrates.

**Table 1:** Precision and robustness metrics for TBZ SERS analysis including descriptive statistics for repeatability and batch-to-batch precision. Intra-day (10 random spots on the same substrate) and inter-day (separate batches) precision at 10 ppm TBZ (RSD%). Robustness: effect of laser power variation (± 1.5 mW) on TBZ quantification (recoveries % and RSD)

Test	Sample	Real (ppm)	Found (ppm)	Recovery (%)	Average intensity at 1004 cm^− 1^	RSD% of individual measurement	RSD% of inter/intra-day
Intra-day precision	**10 random sites**	10.00	10.26	102.6	1908 ± 188	9.85	9.85
Inter-day precision	**batch 1**	10.00	8.984	89.84	1820 ± 116	6.37	2.34
	**batch 2**	10.00	10.21	102.1	1905 ± 116	6.08	
	**batch 3**	10.00	9.842	98.42	1880 ± 89	4.72	
Robustness	**13.5**	10.00	8.975	89.75	1819		3.54
	**15**	10.00	8.745	87.45	1802		
	**16.5**	10.00	10.49	104.9	1923		

Accuracy in analytical methods refers to the capacity to assess the true concentration of an analyte by comparing the measured amount to a known added quantity [35]. To assess the accuracy of SERS-based detection of TBZ, recovery studies were conducted using the standard addition method at three different concentration levels: 75%, 100%, and 125%. In this methodology, established amounts of TBZ standard were spiked into pre-analyzed samples, and the outcomes were compared with predicted values to evaluate recovery efficiency. Recovery calculations are essential in determining the practical use of the SERS substrate, confirming that the method quantifies TBZ reliably, with minimal loss or interference. The results, summarized in Table S3, demonstrate that recovery rates range from 88.8% to 109%, with RSD between 7.5% and 10.4%, reflecting high reliability and minimal deviation from expected results.

**Fig. 5:**
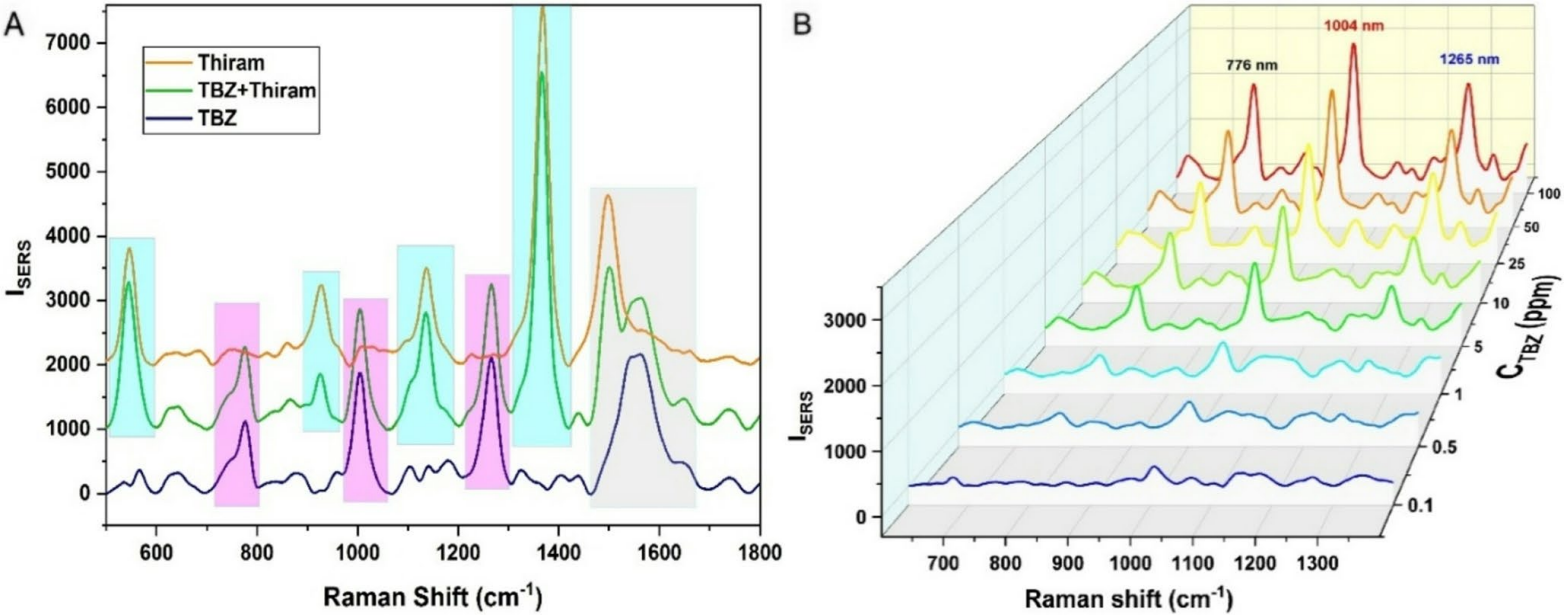
(**A**) Selectivity test: SERS spectra of TBZ (10 ppm), Thiram (10 ppm), and the solution containing 10 ppm of each, (**B**) SERS spectra of TBZ in apple juice (0.1–100 ppm)

The evaluation of selectivity in the SERS method for detecting TBZ involved analyzing potential interferents, particularly thiram, which is a common pesticide used alongside TBZ in agricultural applications. Thiram is a dithiocarbamate compound rich in sulfur atoms, making it structurally similar to TBZ and capable of competing for adsorption sites on the gold nanoparticle surface. A competitive adsorption experiment was performed by preparing a methanolic solution containing 10 ppm TBZ with 10 ppm thiram and analyzing the resulting SERS spectrum. The Raman spectra illustrated in Fig. 5A indicate that when TBZ and thiram were mixed, thiram did not significantly disrupt the TBZ signals. Key Raman peaks associated with TBZ at 776, 1265, and notably 1004 cm^− 1^ remained clearly identifiable with high spectral resolution, showing minimal interference from thiram. Although there was some minor spectral overlap in the region of 1450–1750 cm^− 1^, it did not hinder the identification of TBZ. Fu et al. adapted this strategy earlier. However, they found that thiram and TBZ spectra overlapped between 700 and 1550 cm^− 1^, affecting thiram recoveries in apple samples. However, TBZ could still be detected at 1593 cm^− 1^, with higher TBZ concentrations reducing interference from thiram [36].

In our study, the recovery rate and RSD% of the TBZ SERS signal in the mixture were found to be 94.7% and 13.1%, respectively, compared to values of 97% and 7.7% for TBZ in isolation (Table S3). The mean TBZ signal intensity differed by less than 1% between the two conditions, and Welch’s t-test showed no statistically significant difference between TBZ alone and the TBZ-thiram mixture, *p* = 0.898. These results indicate that the presence of 10 ppm thiram did not produce a statistically significant interference in TBZ quantification. The specificity of the method is further reinforced by the successful detection of TBZ in untreated apple juice (please see later in Sect. 3.6). The complex juice matrix contains polyphenols, sugars, and organic acids; however, these components did not interfere with the main TBZ signals, which remained fully detectable even with reduced intensity. Together, the thiram-addition experiment and the successful detection in untreated apple juice support the practical selectivity of the AuNPs@filter platform under chemically and matrix-relevant conditions. Nevertheless, broader interference studies involving structurally related benzimidazole fungicides and additional co-existing agrochemicals would further strengthen the analytical applicability of the method and remain an important direction for future work.

Robustness was evaluated by assessing the effect of parameter variations, specifically laser power, on the method’s response. Therefore, a ± 1.5 mW variation in laser power was tested at the same spot. The results in Table 1 indicate a slight effect, with an RSD of 3.54% and recoveries ranging from 87.45% to 104.9%, demonstrating that the method is robust and not significantly affected by minor parameter changes.

### TBZ in apple juice analysis

To assess the practical usability of the proposed detection method, a sample of commercially available apple juice was sourced from a local market and analyzed for the detection of TBZ utilizing the AuNPs@filter substrate. Figure 5B illustrates the average SERS spectra (*n* = 8) of TBZ solutions at different concentrations within the apple juice matrix. The characteristic Raman peaks of TBZ were detected, displaying slight spectral variations, along with a decrease in intensity to approximately 73–84% when compared to reference solutions in methanol. This reduction in intensity is likely due to the influence of inherent apple juice components, such as carbohydrates, organic acids, and polyphenols, which may cause competitive adsorption or matrix effects that modify the molecular environment surrounding TBZ [37].

To visualize matrix effects, we first recorded the SERS spectrum of blank apple juice on the AuNPs@filter interface (Figure S9). The juice matrix produces broad bands but no sharp features at the TBZ diagnostic positions (776, 1004, 1265 cm^− 1^). After subtraction of this juice background from the spectrum of apple juice spiked with 10 and 100 ppm TBZ, the TBZ peaks emerge clearly, confirming that the juice components mainly contribute to a smooth background that can be efficiently removed by the preprocessing step. Quantification in apple juice was therefore performed using matrix-matched standards and the same baseline-correction protocol as detailed in the Experimental Section.

**Fig. 6:**
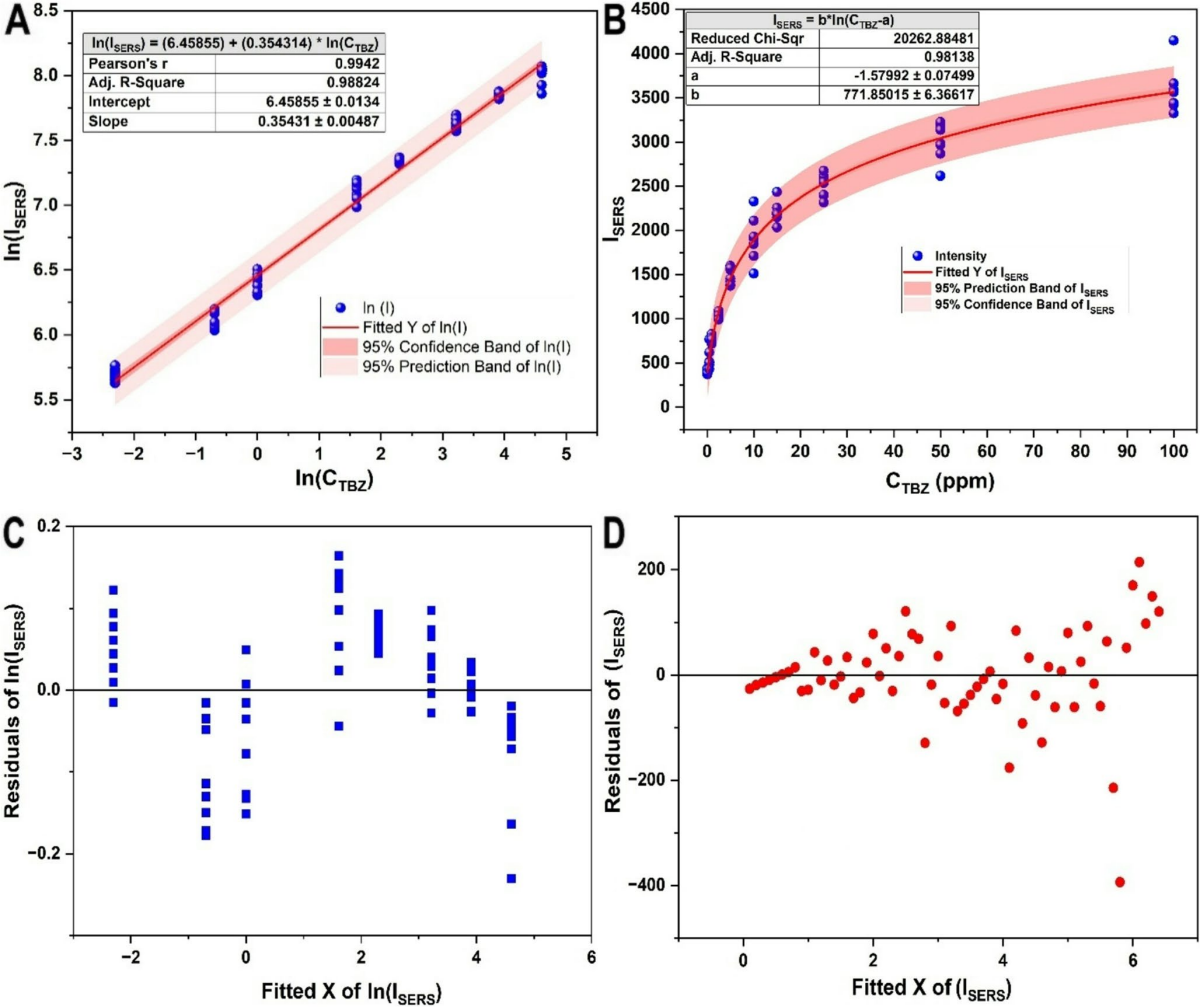
(**A**) Double-ln calibration plot in apple juice showing linearized fit, (**B**) nonlinear logarithmic model fit, and residuals plot for (**C**) linearized and (**D**) nonlinear models

The calibration plots for the Raman peak at 1004 cm^− 1^ (shown in Fig. 6A, B) demonstrate strong correlation coefficients for both linearized and nonlinear fitting approaches for the analysis of untreated apple juice samples. Upon comparing the fitting methods, the nonlinear logarithmic model is shown to provide greater predictive accuracy again. This finding is supported by higher R² values, decreased standard deviation, and lower RSD% when compared to the linearized model, as outlined in Table 2. The advantages of the nonlinear model are further validated through residual analysis (Fig. 6C, D), which reveals a more uniform distribution of residuals and diminished systematic errors, suggesting a closer alignment with the experimental data. Consequently, the nonlinear logarithmic model is once again deemed the more suitable option for quantifying TBZ in apple juice. The TBZ-spiked apple juice samples analyzed by the nonlinear calibration model showed satisfactory recoveries, with Predicted C/Real C values ranging from 82.9% to 105% across the investigated concentration levels (Table 2), further demonstrating good quantitative accuracy of the method in the real juice matrix.

**Table 2:** TBZ calibration in apple juice matrix. Predicted vs. real TBZ concentrations using linearized and nonlinear models

	Linearized fit				Nonlinear fit			
Real C (ppm)	Predicted C (ppm)	Predicted C/Real C (%)	±SD	RSD%	Predicted C (ppm)	Predicted C/Real C (%)	±SD	RSD%
0.100	0.115	115	20.2	19.9	0.0829	82.9	7.72	7.82
0.500	0.371	74.2			0.504	100		
1.00	0.845	84.5			1.04	104		
5.00	6.40	128			5.25	105		
10.0	12.1	121			9.46	94.6		
25.0	27.6	110			23.2	92.8		
50.0	50.4	100.8			51.3	103		
100	79.3	79.3			104	104		

Furthermore, by using the same methodology applied to the methanolic standard solutions, a LOD of 0.10 ppm was determined in apple juice (Figure S10). This value is very close to that is achieved in methanol standards (LOD: 0.08 ppm), which illustrates the robustness of the method in terms of its ability to withstand potential matrix interferences while maintaining strong interface-driven signal amplification. Although direct comparison between a solution-based SERS LOD and commodity-specific MRLs should be made cautiously, the obtained LOD is well below typical regulatory limits reported for TBZ on fruit commodities. For example, EFSA and related summaries report MRLs of about 4 mg/kg for pome fruits (including apples) and up to 7 mg/kg for citrus fruit and mangoes [32]. This supports the practical relevance of the method for residue screening in apple juice and other fruit-based products.

### Comparative analysis and advantages of the proposed method

The critical review of SERS-based approaches reported in the literature for TBZ detection in Table S4 (provided in the ESM) highlights several notable practical limitations, such as complicated synthesis protocols, dependence on surface functionalization (e.g., surfactants, thiols, polymers), and labor-intensive sample processing techniques (e.g., centrifugation, salting-out, QuEChERS: Quick, Easy, Cheap, Effective, Rugged, and Safe). Additionally, many approaches require sophisticated chemometric tools (like SNV: Standard Normal Variate, PLS: Partial Least Squares, PLSR: PLS Regression, PCA: Principal Component Analysis, RFE-RF: Recursive Feature Elimination with Random Forest, SVM: Support Vector Machine, DFT: Density Functional Theory, SVR: Support Vector Regression) for data analysis, which, although effective, can limit accessibility and practical application. While low detection limits are frequently reported, they often come at the cost of simplicity, reproducibility, or affordability. Conversely, our study introduces a practical and analytically relevant method, featuring a straightforward nano-interface fabrication strategy without the need for functionalization or intricate nanostructures. This approach requires no sample pretreatment and circumvents the necessity of chemometric modelling, enabling straightforward data analysis driven by the strong signal amplification of the engineered interface. Crucially, it achieves a reliable LOD suitable for regulatory oversight. Collectively, these features make the proposed three-dimensional plasmonic interface a robust, user-friendly, and field-applicable solution for TBZ detection.

## Conclusion

This work presents a simple, solvent-free and scalable route to fabricate paper-based SERS interfaces by directly depositing spark-ablation-generated Au nanoparticles onto cellulose filters. The resulting AuNPs@filter substrate forms a 3D fibrous, percolated plasmonic network that provides reproducible SERS response and robust performance under realistic measurement conditions. The platform enabled quantitative TBZ detection in methanol and untreated commercial apple juice over 0.1–100 ppm using a nonlinear logarithmic calibration model, avoiding advanced chemometric processing while retaining high predictive accuracy. ICH Q2(R2)-guided validation, supported by additional statistical analysis, confirmed repeatability, precision, accuracy, selectivity against thiram, and robustness to moderate laser-power variations. The LOD (IUPAC/ICH 3.3σ/S) was 0.08 ppm in methanol and 0.10 ppm in untreated apple juice, supporting practical residue monitoring without labor-intensive sample preparation or surface functionalization. Overall, AuNPs@filters offer a green, cost-effective and field-deployable SERS strategy linking spark-based nanomanufacturing with applied food-safety and chemical/biosensing analysis. Rather than maximizing sensitivity alone, the method offers a balanced combination of green fabrication, straightforward calibration, and applicability in a real food matrix. Further improvements in spatial uniformity could be pursued in future work by refining the aerosol deposition conditions and exploring gentle surface modifications of the cellulose support to promote more homogeneous nanoparticle binding, while maintaining the simplicity and green character of the fabrication route.

## Supplementary Information

Below is the link to the electronic supplementary material.

Supplementary Material 1 (DOCX 1.41 MB)

## Data Availability

The data supporting the findings of this study are available from the corresponding author upon reasonable request.
