# Supplementary material for: Gas-phase engineered gold-on-paper SERS substrates for quantitative thiabendazole sensing in real matrices
Source: Mikrochim Acta. 2026 Jul 1;193(7):505. doi: 10.1007/s00604-026-08230-0 (PMC13323355; doi:10.1007/s00604-026-08230-0)
Supplement: Supplementary file 1 — Supplementary Material 1 (DOCX 1.41 MB) [file 604_2026_8230_MOESM1_ESM.docx]

**Electronic Supplementary Material**

**for**

**Gas-phase engineered gold-on-paper SERS substrates for quantitative thiabendazole sensing in real matrices**

Maher Darwish^1,2^, Viktória Horváth^1^, Hanan Mohammad^3^, Gábor Katona^3^, Judit Kopniczky^1^, Zsolt Geretovszky^1^ and Attila Kohut^1*^

*^1^Department of Optics and Quantum Electronics, University of Szeged, Dóm sq. 9, 6720 Szeged, Hungary*

*^2^Department of Pharmaceutical Chemistry and Drug Control, Faculty of Pharmacy, Wadi International University, Homs, Syria*

*^3^Institute of Pharmaceutical Technology and Regulatory Affairs, Faculty of Pharmacy, University of Szeged, H-6720 Szeged, Hungary*

*****Corresponding author, email: kohut.attila@szte.hu

# **Experimental Section**

## Materials, reagents and instrumentation

All chemicals employed in this research were of analytical reagent grade. TBZ and thiram were sourced from Sigma-Aldrich; Merck KGaA. Anhydrous methanol (99.9%) was acquired from VWR Chemicals BDH. Rhodamine 6G (R6G, 99%) was obtained from ACROS Organics. Images from the scanning electron microscope (SEM) were captured using a Hitachi S-4700 to evaluate the morphological characteristics of the engineered nano-interfaces. Ultraviolet-visible (UV-Vis) absorption measurements were conducted utilizing a Shimadzu UV-2101PC spectrophotometer to confirm the plasmonic resonance properties of the substrates.


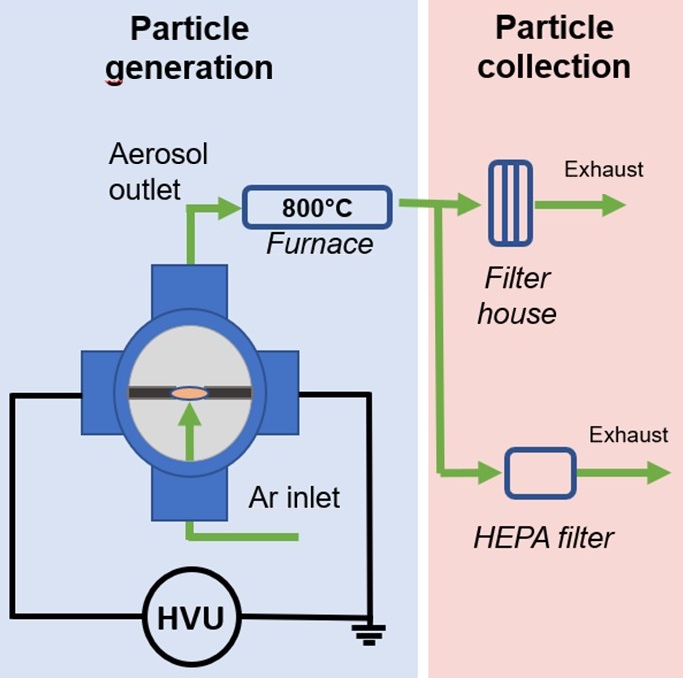


Figure S1. Schematic representation of the setup used for the SERS substrate fabrication.

## Statistical analysis.

Statistical analyses were performed using R software. Data are reported as mean ± standard deviation (SD), and relative standard deviation (RSD%) was calculated as SD/mean × 100. Group-wise statistical comparisons were used to determine whether observed SERS intensity variations were statistically significant. One-way ANOVA was applied for comparisons among multiple groups, Welch’s t-test for two-group comparisons, and Kruskal-Wallis testing as a non-parametric confirmatory test when appropriate. A p-value < 0.05 was considered statistically significant.

**
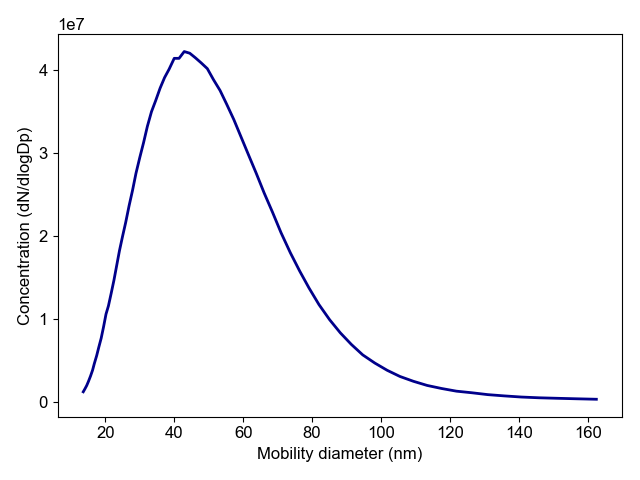
**

Figure S2. Electrical mobility size distribution of heat-treated 50 at. % gold-silver alloy nanoparticles measured in nitrogen, after 10× dilution by means of a Scanning Mobility Particle Sizer (SMPS, Model 3082 classifier, Model 3077A neutralizer, Model 3756 particle counter, TSI Inc.).


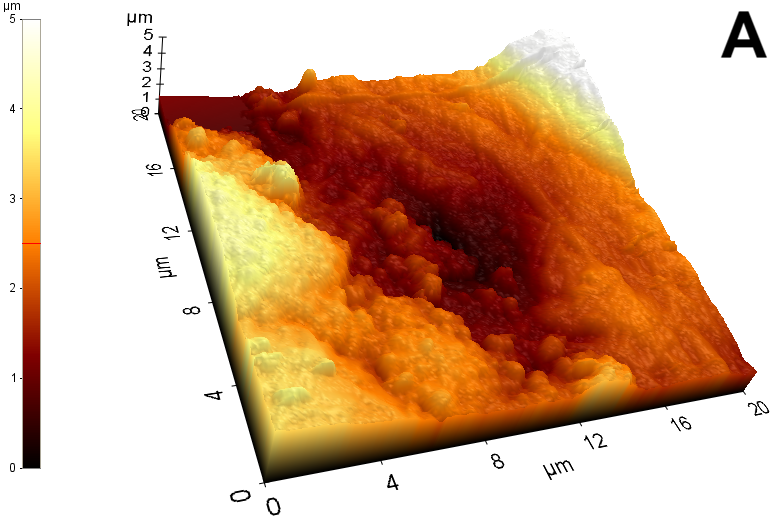

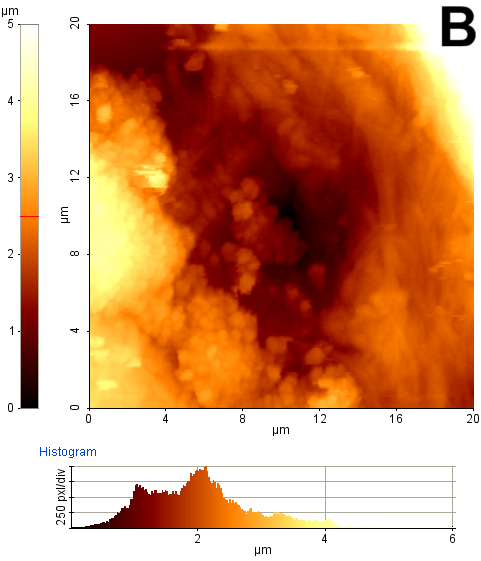


Figure S3. AFM topography images of the AuNPs@filter substrate. AFM scans recorded over (A) 20×20 µm^2^, together with (B) the corresponding 2D AFM image and height histogram.


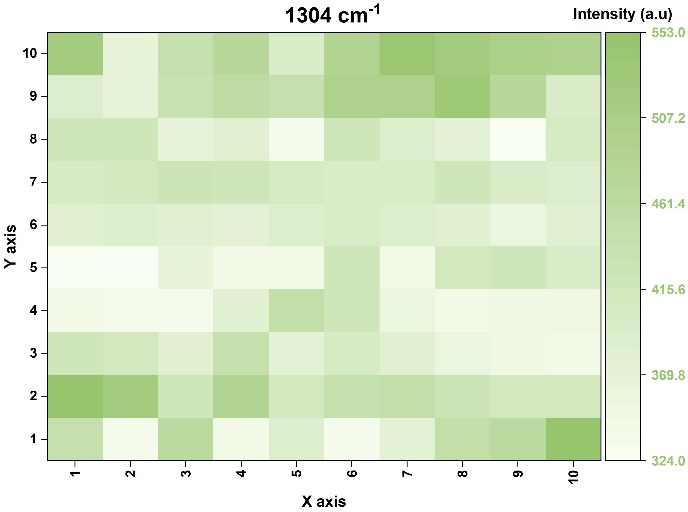

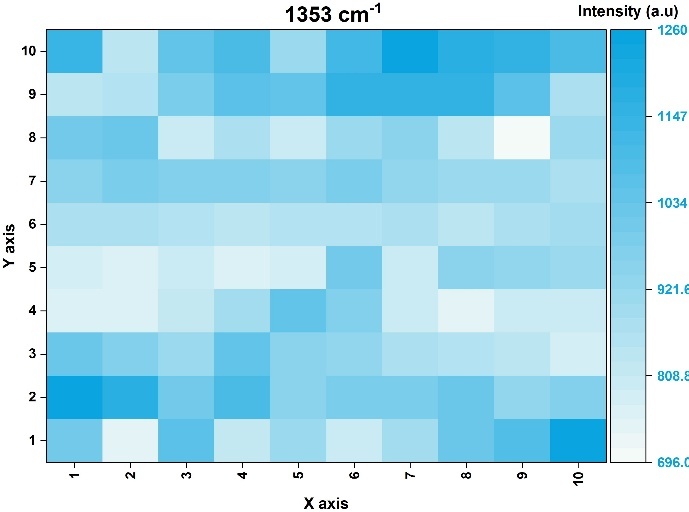

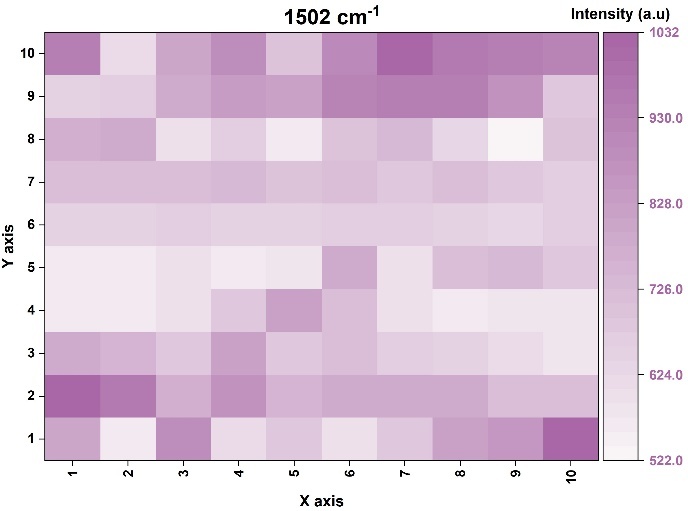


Figure S4. Spatial intensity distribution maps for the other three characteristic R6G Raman bands. The color gradients reflect local variations in signal intensity, directly correlating with the heterogeneous distribution of gold nanoparticle clusters formed during aerosol deposition.


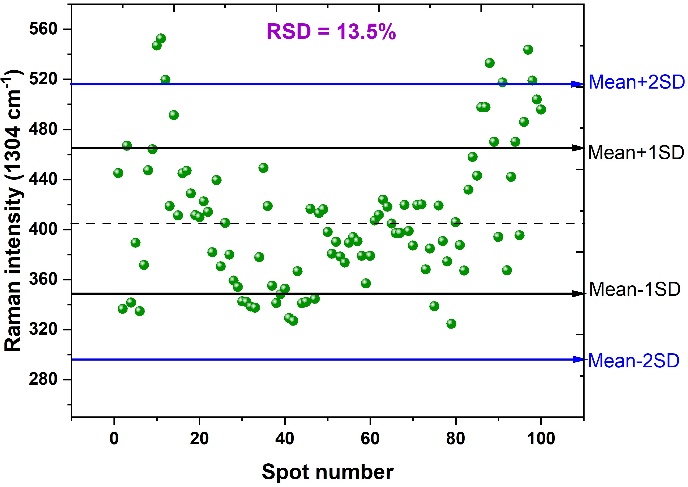

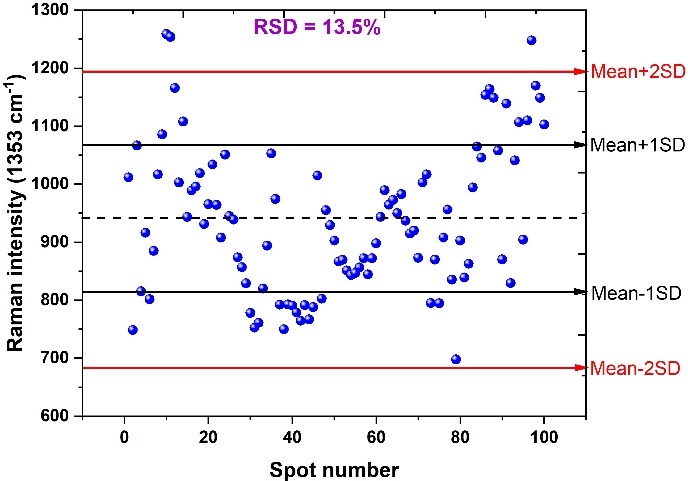

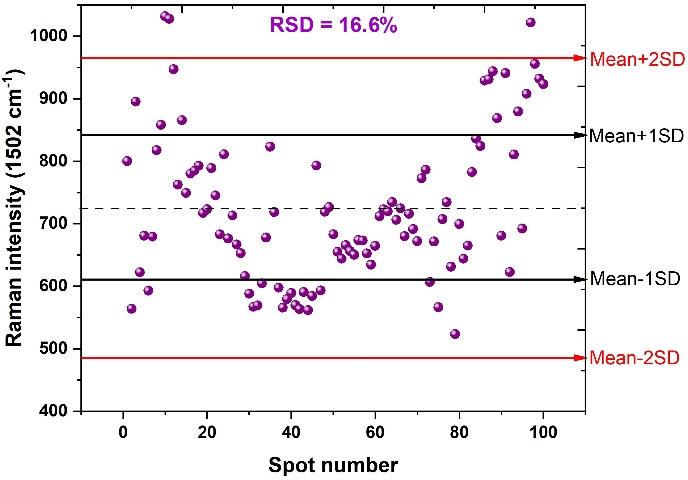


Figure S5. Representative intensity scatter plots for the other characteristic R6G Raman bands.

## The analytical enhancement factor calculation

The analytical enhancement factor (AEF) of the substrates was estimated using R6G as the reporter molecule. The 1502 cm^-1^ band was selected because it was the R6G band clearly detectable in both the SERS spectrum and the normal Raman reference spectrum. The SERS intensity of this band obtained from 100 µM R6G on the AuNPs@filter substrate was compared with the normal Raman intensity of the same band from 10 mM R6G on bare Whatman filter paper under identical instrumental conditions. The AEF was calculated according to:

$$AEF=\left( \frac{I_{SERS}}{I_{Raman}} \right)\times\left( \frac{C_{Raman}}{C_{SERS}} \right)$$

The estimated AEF of approximately 1.4 × 10^3^ confirms that the AuNP-cellulose interface provides measurable Raman signal amplification and is of the same order of magnitude as the value previously reported for our analogous spark-ablation-deposited Au/Ag nanoparticle substrates on filter paper [1]. The relatively low AEF value characteristic to the AuNPs@filter substrates illustrates well that the AEF does not necessarily a good predictor of a SERS substrates performance in a specific application. As our results prove, a low LOD and good recovery rate can be achieved even with a relatively small AEF in a real-world application. This strengthens the fact that analytical SERS applications should not rely on a single, often ambiguously determined metric, such as EF or AEF [2].

Table S1. Descriptive statistics and quadrant-wise comparison of the 100-point R6G SERS mapping dataset on the AuNPs@filter substrate.

| **Raman band / cm^-1^** | **n** | **Mean intensity / a.u.** | **SD / a.u.** | **RSD%** | **Mean ± 1SD / a.u.** | **Mean ± 2SD / a.u.** | **One-way ANOVA p-value** | **Kruskal–Wallis p-value** |
| --- | --- | --- | --- | --- | --- | --- | --- | --- |
| 1184 | 100 | 470.1 | 67.2 | 14.30 | 402.9–537.3 | 335.7–604.5 | 0.639 | 0.563 |
| 1304 | 100 | 408.5 | 55.2 | 13.51 | 353.3–463.7 | 298.1–518.9 | 0.129 | 0.186 |
| 1354 | 100 | 938.9 | 127.0 | 13.52 | 811.9–1065.9 | 684.9–1192.9 | 0.333 | 0.387 |
| 1502 | 100 | 724.6 | 120.1 | 16.57 | 604.5–844.7 | 484.4–964.8 | 0.226 | 0.366 |

The 10 × 10 mapping grid was divided into four equal quadrants for quadrant-wise comparison. One-way ANOVA was used to test for large-area differences among quadrants, while the Kruskal–Wallis test was used as a non-parametric confirmatory test. p > 0.05 indicates no statistically significant quadrant-dependent intensity variation.

Table S2. Calibration performance for TBZ in methanol. Comparison of predicted vs. actual TBZ concentrations (ppm) using the linearized (ln-ln) model and the nonlinear model.

|  | **Linearized fit** | | | | **Nonlinear fit** | | | |
| --- | --- | --- | --- | --- | --- | --- | --- | --- |
| **Real C (ppm)** | **Predicted C (ppm)** | **Predicted C/Real C (%)** | **±SD^*^** | **RSD%** | **Predicted C (ppm)** | **Predicted C/Real C (%)** | **±SD** | **RSD%** |
| 0.100 | 0.136 | 136 | 24.1 | 23.2 | 0.109 | 109 | 7.48 | 7.43 |
| 0.500 | 0.323 | 64.7 |  |  | 0.443 | 88.7 |  |  |
| 1.00 | 0.881 | 88.1 |  |  | 1.12 | 112 |  |  |
| 2.50 | 2.16 | 86.3 |  |  | 2.28 | 91.0 |  |  |
| 5.00 | 5.89 | 118 |  |  | 5.13 | 103 |  |  |
| 10.0 | 12.7 | 127 |  |  | 10.3 | 103 |  |  |
| 15.0 | 19.3 | 129 |  |  | 15.8 | 106 |  |  |
| 25.0 | 28.0 | 112 |  |  | 24.1 | 96.6 |  |  |
| 50.0 | 48.3 | 96.6 |  |  | 48.5 | 96.9 |  |  |
| 100 | 78.9 | 78.9 |  |  | 101 | 101 |  |  |

^*^SD=Standard deviation


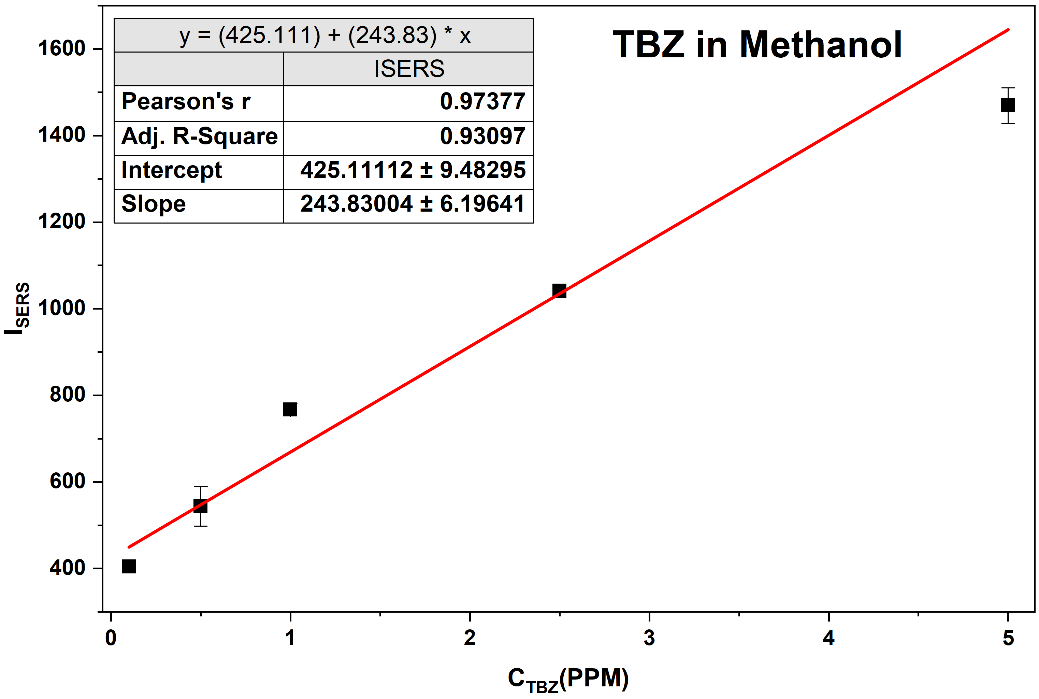


Figure S6. Linear regression plot for SERS detection of TBZ in methanol on AuNPs@filter substrate. SERS intensity at 1004 cm^-1^ is plotted against TBZ concentration (0.1-5 ppm).


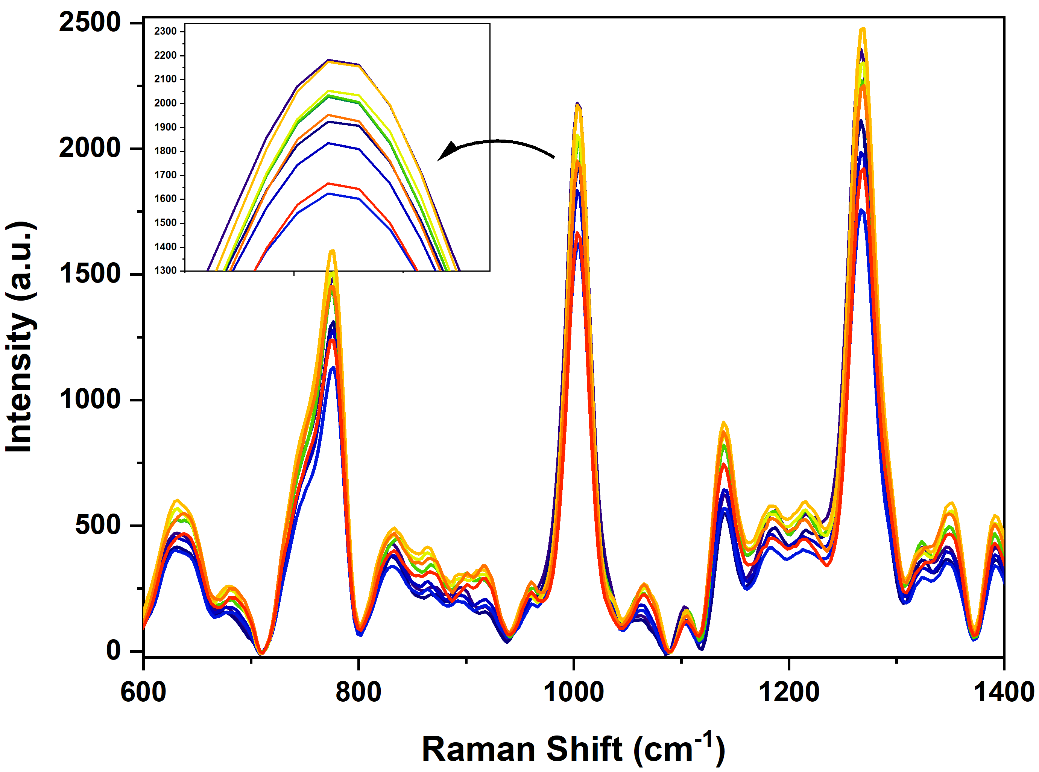


Figure S7. Overlay of ten baseline‑corrected SERS spectra of 10 ppm TBZ in methanol collected from random sites on a single AuNPs@filter substrate, demonstrating intra‑day repeatability. Inset: Magnified view of the 1004 cm^-1^ peak region showing the intensity distribution among the ten replicates.


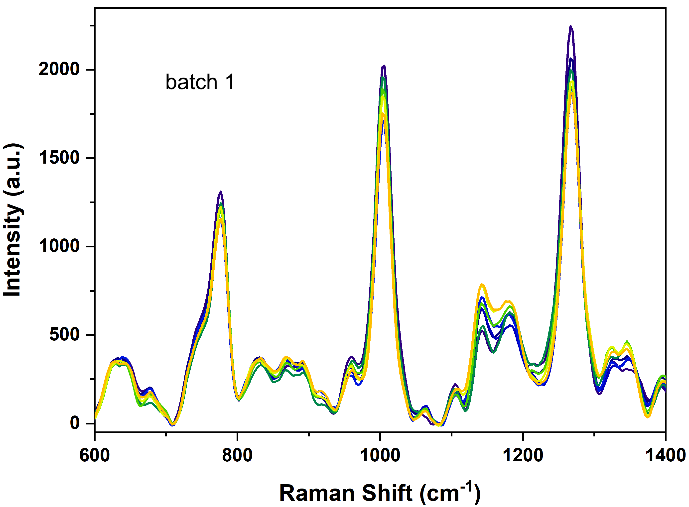

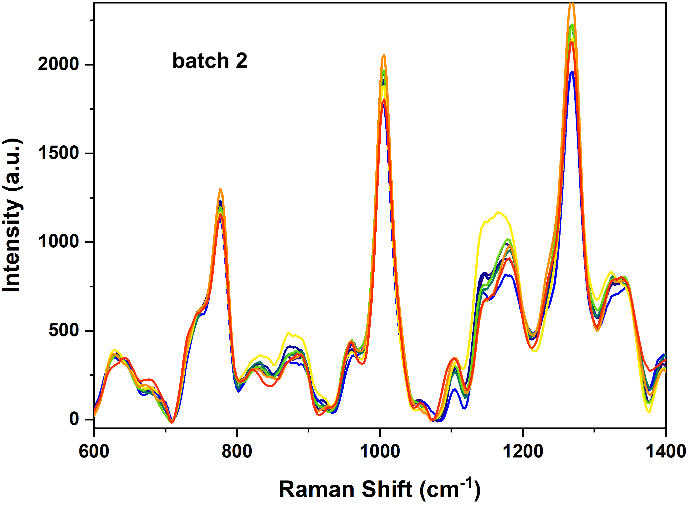

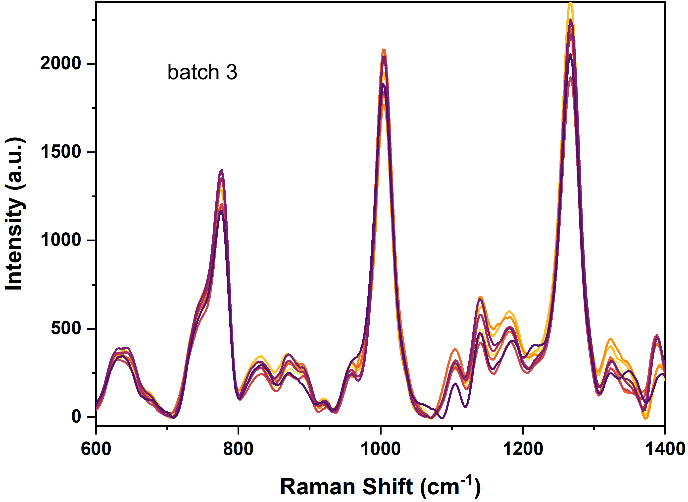


Figure S8. Inter‑day precision of the SERS method for TBZ detection.

Overlay of baseline‑corrected SERS spectra of 10 ppm TBZ in methanol measured on three independently fabricated SERS substrates (Patch 1, 2, and 3) prepared on different days. The characteristic TBZ peak at 1004 cm^-1^ is visible and consistent across all three batches, demonstrating excellent batch‑to‑batch reproducibility.

Table S3. Recovery performance of the AuNPs@filter SERS method for TBZ quantification using the standard addition approach (accuracy) and mixing with thiram (selectivity).

| **Study** | | **Real C (ppm)** | **Predicted C (ppm)** | **Recovery (%)** | **RSD (%)** |
| --- | --- | --- | --- | --- | --- |
| **Accuracy** | | 7.50 | 6.66 | 88.8 | 7.50 |
|  |  | 10.0 | 9.70 | 97.0 | 7.70 |
|  |  | 12.5 | 13.6 | 109 | 10.4 |
| **Selectivity** | TBZ alone | 10.0 | 9.70 | 97.0 | 7.70 |
|  | TBZ in Mixture | 10.0 | 9.47 | 94.7 | 13.1 |


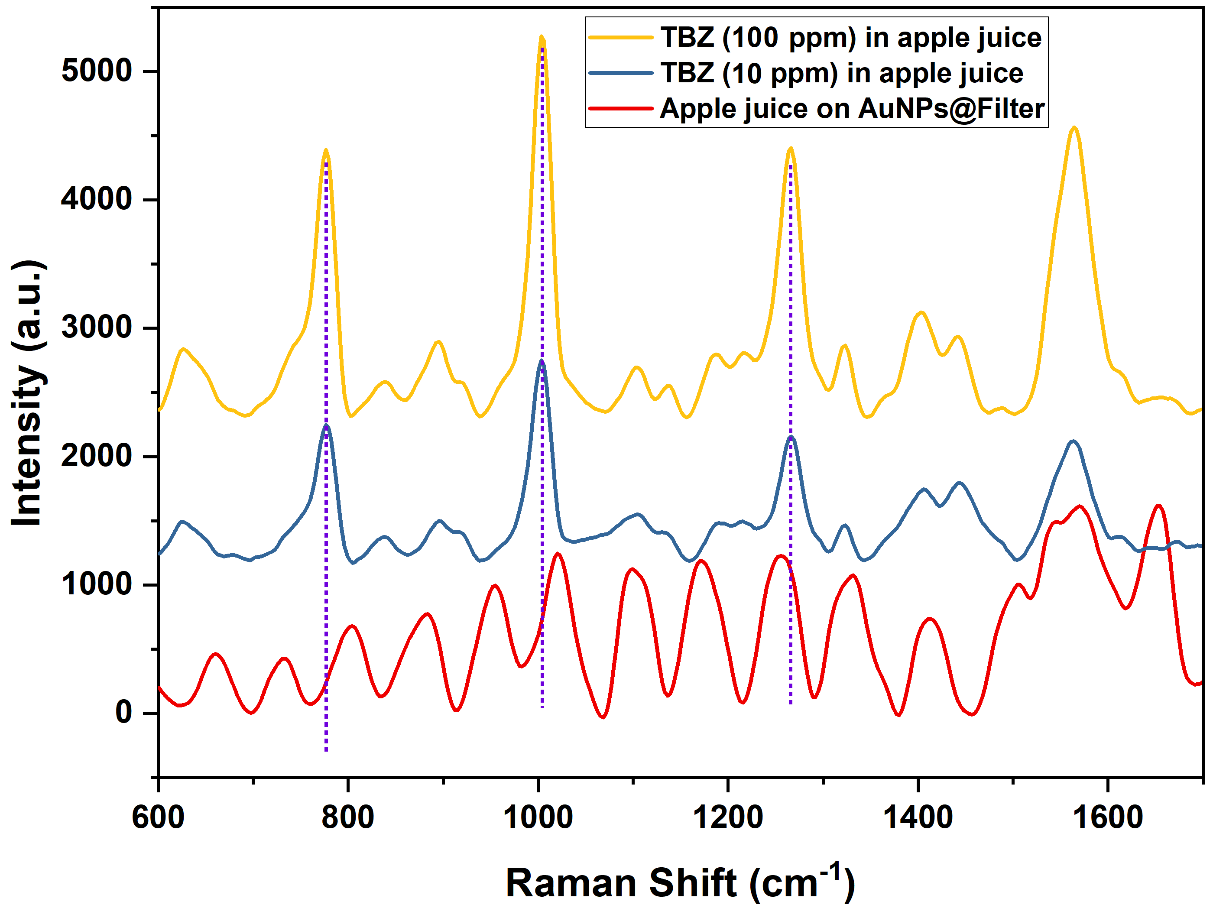


Figure S9. SERS spectrum of blank apple juice on AuNPs@filter (red) and background‑subtracted SERS spectra of apple juice spiked with TBZ at 10 ppm (blue) and 100 ppm (yellow). The vertical dashed lines indicate the TBZ diagnostic bands at 776, 1004 and 1265 cm⁻¹, which remain clearly visible after subtraction of the juice background.


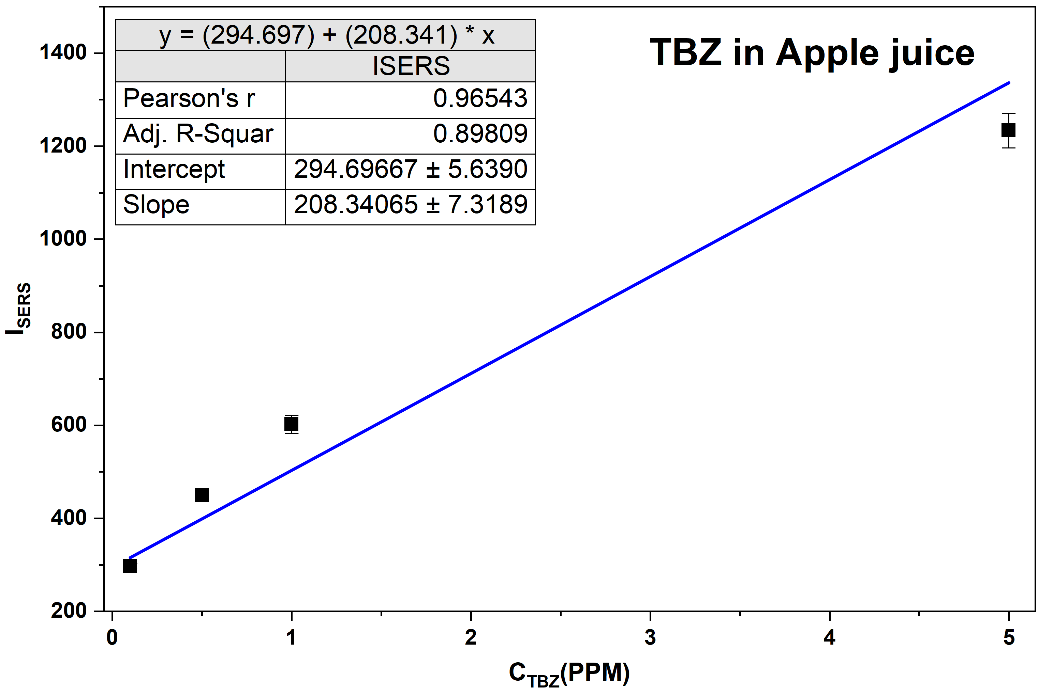


Figure S10. Linear regression plot for SERS detection of TBZ in apple juice on AuNPs@filter substrate. SERS intensity at 1004 cm^-1^ is plotted against TBZ concentration (0.1-5 ppm) in untreated apple juice.

Table S4. Survey of reported SERS methods for TBZ detection and their limitations.

| **SERS Substrate** | **Synthesis Method** | **Functionalization** | **Sample Matrix** | **Sample Pretreatment** | ***LOD (ppm unless indicated by ‡ = ng/cm^2^)*** | **Chemometrics** | **Reference** |  |
| --- | --- | --- | --- | --- | --- | --- | --- | --- |
| Au NPs | Turkevich method | None | Soil extract | Centrifugation | 0.1 | SNV, PLS | [3] |  |
| Au@Ag NPs | Turkevich +Ascorbic reduction | None | Water, juice, serum | Filtration | (2.95-4.94) × 10^-5^ | None | [4] |  |
| Au@Ag NPs | Chemical reduction + Seed-mediated growth | None | Apple juice | Salting-out | 0.05 | None | [5] |  |
| Au@Ag-2ME NPs | Chemical reduction + Seed-mediated growth | 2-Mercaptoethanol (2-ME) | Apple puree | QuEChERS | 0.0064 | None | [6] |  |
| Au@Ag NPs Array | Chemical reduction + Seed-mediated growth | poly(methylmethacrylate)/ qPCR film | Mixed juices | None | 0.021–0.069 | None | [7] |  |
| Au@Ag-TGA NPs | Chemical reduction + Seed-mediated growth | Thioglycolic acid (TGA) | Milk | pH adjustment, Centrifugation | 0.12 | None | [8] |  |
| Au@Ag NPs | Chemical reduction + Seed-mediated growth | None | Apple juice | QuEChERS | 0.06 | None | [9] |  |
| Au@Ag NPs | Chemical reduction + Seed-mediated growth | None | Apple juice | Salt-assisted extraction | 0.1 | RFE-RF | [10] |  |
| Au nanorods array | Modified seed-mediated | cetyltrimethylammonium bromide | Apples | Centrifugation | 0.06 | PLSR | [11] |  |
| Au nanorods array | Modified seed-mediated | cetyltrimethylammonium bromide | Juice samples | Centrifugation | 0.149–0.216 | PLS | [12] |  |
| Ag-Au@MOF | In-situ chemical reduction | IP6-functionalized metal organic framework (MOF) | Peach juice | None | 0.05 | None | [13] |  |
| AgNPs@Cellulose Acetate | In-situ chemical reduction | None | Apple peels | None | 15.1‡ | None | [14] |  |
| Flower-like AgNPs | Chemical reduction | Polyvinylpyrrolidone | Juice samples | Filtration, Salting-out, Centrifugation | 0.24 | RFE, SVM | [15] |  |
| Ag colloids | Chemical reduction | None | Bananas & Citrus | None | 78 | DFT | [16] |  |
| Ag colloids | Chemical reduction | Molecularly imprinted polymer (MIP) | Orange juice | MIP solid-phase extraction | 4 | None | [17] |  |
| Ag colloids | Chemical reduction | None | Blueberry extract | Bentonite filtration | 0.023 | None | [18] |  |
| AgNPs in Agar gel | Chemical reduction | None | Apple peels | None | 200‡ | None | [19] |  |
| Au colloids | Chemical reduction | None | Apple extracts | QuEChERS | 100 | PLSR | [20] |  |
| Fe₃O₄@SiO₂@Ag-SH | Chemical reduction | SH-Fe₃O₄@SiO₂ | Corn | Filtration, Centrifugation | 0.093 | PLSR, SVR | [21] |  |
| Ag dendrites | Replacement reaction | None | Apple surface | None | 0.01 | PCA, PLS | [22] |  |
| AgNP film | Convective self-assembly | None | Standard solution | None | 0.047 | PCA | [23] |  |
| Au nanofinger | Nanoimprint lithography | None | Apple skin | None | 0.007 | None | [24] |  |
| Fe₃O₄@GO@Ag | Layer-by-layer assembly | Fe₃O₄@Graphene oxide (GO) | Apple peels | None | 40‡ | None | [25] |  |
| **Au NPs** | **Spark ablation** | **None** | **Apple juice** | **None** | **0.1** | **None** | **This study** |  |
| ‡ Values expressed as ng/cm^2^ correspond to surface loadings reported directly on solid samples (e.g., fruit peels) in the original reference. For all other entries, LOD is given as solution concentration (ppm) of the analyte before deposition on the SERS substrate. | | | | | | | | |

**References**

[1] V. Horváth, D. Megyeri, J. Kopniczky, M. Darwish, Z. Geretovszky, A. Kohut, Spark Ablation-Generated Nanoparticles on Filter Paper: A 3D SERS Platform for Cost-Effective and Rapid Thiram Sensing, ACS Applied Nano Materials 8(37) (2025) 17934-17951.

[2] S.E.J. Bell, G. Charron, E. Cortés, J. Kneipp, M.L. de la Chapelle, J. Langer, M. Procházka, V. Tran, S. Schlücker, Towards Reliable and Quantitative Surface-Enhanced Raman Scattering (SERS): From Key Parameters to Good Analytical Practice, Angewandte Chemie International Edition 59(14) (2020) 5454-5462.

[3] P. Nie, T. Dong, S. Xiao, L. Lin, Y. He, F. Qu, Quantitative Determination of Thiabendazole in Soil Extracts by Surface-Enhanced Raman Spectroscopy, Molecules 23(8) (2018) 1949.

[4] H. Park, G. Kim, W. Kim, E. Park, J. Park, J. Park, Highly Sensitive and Wide-Range Detection of Thiabendazole via Surface-Enhanced Raman Scattering Using Bimetallic Nanoparticle-Functionalized Nanopillars, Biosensors 14(3) (2024) 133.

[5] X. Li, Y. Zhang, M. Awais, H. Zhang, S.M.Z.A. Naqvi, L. Li, Y. Xiong, J. Hu, Analysis and experimental assessment of an optimized SERS substrate used to detect thiabendazole in apples with high sensitivity, Analytical and Bioanalytical Chemistry 416(2) (2024) 497-508.

[6] N. Hussain, H. Pu, D.-W. Sun, Synthesis of bimetallic core-shelled nanoparticles modified by 2-mercaptoethanol as SERS substrates for detecting ferbam and thiabendazole in apple puree, Food Additives & Contaminants: Part A 38(8) (2021) 1386-1399.

[7] K. Wang, D.-W. Sun, H. Pu, Q. Wei, L. Huang, Stable, Flexible, and High-Performance SERS Chip Enabled by a Ternary Film-Packaged Plasmonic Nanoparticle Array, ACS Applied Materials & Interfaces 11(32) (2019) 29177-29186.

[8] A. Hussain, H. Pu, B. Hu, D.-W. Sun, Au@Ag-TGANPs based SERS for facile screening of thiabendazole and ferbam in liquid milk, Spectrochimica Acta Part A: Molecular and Biomolecular Spectroscopy 245 (2021) 118908.

[9] Y. Song, H. Qiu, Y. Huang, X. Wang, K. Lai, Rapid detection of thiabendazole residues in apple juice by surface-enhanced Raman scattering coupled with silver coated gold nanoparticles, Spectrochimica Acta Part A: Molecular and Biomolecular Spectroscopy 303 (2023) 123189.

[10] X. Li, Y. Zhang, M. Awais, S.M. Zaigham Abbas Naqvi, L. Li, H. Chen, J. Hu, Exploration of efficient SERS features extraction algorithm for rapid detection of thiabendazole residues in apples, LWT 187 (2023) 115310.

[11] G. Fu, D.-W. Sun, H. Pu, Q. Wei, Fabrication of gold nanorods for SERS detection of thiabendazole in apple, Talanta 195 (2019) 841-849.

[12] F.K. Alsammarraie, M. Lin, A. Mustapha, H. Lin, X. Chen, Y. Chen, H. Wang, M. Huang, Rapid determination of thiabendazole in juice by SERS coupled with novel gold nanosubstrates, Food Chemistry 259 (2018) 219-225.

[13] T. Xuan, Y. Gao, Y. Cai, X. Guo, Y. Wen, H. Yang, Fabrication and characterization of the stable Ag-Au-metal-organic-frameworks: An application for sensitive detection of thiabendazole, Sensors and Actuators B: Chemical 293 (2019) 289-295.

[14] C. Zong, M. Ge, H. Pan, J. Wang, X. Nie, Q. Zhang, W. Zhao, X. Liu, Y. Yu, In situ synthesis of low-cost and large-scale flexible metal nanoparticle–polymer composite films as highly sensitive SERS substrates for surface trace analysis, RSC Advances 9(5) (2019) 2857-2864.

[15] H. Li, X. Luo, S.A. Haruna, W. Zhou, Q. Chen, Rapid detection of thiabendazole in food using SERS coupled with flower-like AgNPs and PSL-based variable selection algorithms, Journal of Food Composition and Analysis 115 (2023) 105016.

[16] C. Müller, L. David, V. Chiş, S.C. Pînzaru, Detection of thiabendazole applied on citrus fruits and bananas using surface enhanced Raman scattering, Food Chemistry 145 (2014) 814-820.

[17] J. Feng, Y. Hu, E. Grant, X. Lu, Determination of thiabendazole in orange juice using an MISPE-SERS chemosensor, Food Chemistry 239 (2018) 816-822.

[18] C. Müller Molnár, C. Berghian-Groșan, D.A. Măgdaș, S. Cîntă Pînzaru, Surface-Enhance Raman Spectroscopy Detection of Thiabendazole in Frozen Food Products: The Case of Blueberries and Their Extracts, Chemosensors 11(9) (2023) 505.

[19] M.L. Rizzato, A.L. Picone, R.M. Romano, A facile method for in-situ detection of thiabendazole residues in fruit and vegetable peels using Surface-Enhanced Raman Spectroscopy, Talanta Open 7 (2023) 100223.

[20] H. Luo, Y. Huang, K. Lai, B.A. Rasco, Y. Fan, Surface-enhanced Raman spectroscopy coupled with gold nanoparticles for rapid detection of phosmet and thiabendazole residues in apples, Food Control 68 (2016) 229-235.

[21] T. Wang, C. Xie, Q. You, X. Tian, X. Xu, Qualitative and quantitative analysis of four benzimidazole residues in food by surface-enhanced Raman spectroscopy combined with chemometrics, Food Chemistry 424 (2023) 136479.

[22] L. He, T. Chen, T.P. Labuza, Recovery and quantitative detection of thiabendazole on apples using a surface swab capture method followed by surface-enhanced Raman spectroscopy, Food Chemistry 148 (2014) 42-46.

[23] I.A. Brezestean, N. Tosa, A. Falamas, D. Cuibus, C.M. Muntean, A. Bende, B. Cozar, C. Berghian-Grosan, C. Farcău, Silver Nanoparticle Films Obtained by Convective Self-Assembly for Surface-Enhanced Raman Spectroscopy Analyses of the Pesticides Thiabendazole and Endosulfan, Frontiers in Chemistry Volume 10 - 2022 (2022).

[24] A. Kim, S.J. Barcelo, Z. Li, SERS-based pesticide detection by using nanofinger sensors, Nanotechnology 26(1) (2015) 015502.

[25] Z. Liu, Y. Wang, R. Deng, L. Yang, S. Yu, S. Xu, W. Xu, Fe3O4@Graphene Oxide@Ag Particles for Surface Magnet Solid-Phase Extraction Surface-Enhanced Raman Scattering (SMSPE-SERS): From Sample Pretreatment to Detection All-in-One, ACS Applied Materials & Interfaces 8(22) (2016) 14160-14168.
